# An Integrated mHealth App for Smoking Cessation in Black Smokers With Anxiety: Protocol for a Randomized Controlled Trial

**DOI:** 10.2196/38905

**Published:** 2022-05-30

**Authors:** Michael S Businelle, Lorra Garey, Matthew W Gallagher, Emily T Hébert, Anka Vujanovic, Adam Alexander, Krista Kezbers, Cameron Matoska, Jillian Robison, Audrey Montgomery, Michael J Zvolensky

**Affiliations:** 1 TSET Health Promotion Research Center University of Oklahoma Health Sciences Center Oklahoma City, OK United States; 2 Department of Family and Preventive Medicine University of Oklahoma Health Sciences Center Oklahoma City, OK United States; 3 HEALTH Institute University of Houston Houston, TX United States; 4 Department of Psychology University of Houston Houston, TX United States; 5 Texas Institute for Measurement, Evaluation, and Statistics University of Houston Houston, TX United States; 6 Department of Health Promotion and Behavioral Sciences UTHealth School of Public Health Austin, TX United States

**Keywords:** smoking cessation, treatment, Black, African American, anxiety sensitivity, mHealth, just-in-time adaptive intervention, mobile phone

## Abstract

**Background:**

Black smokers have greater difficulty in quitting and higher rates of smoking-related diseases and disabilities than the general population. The smoking disparities experienced by this group are, in part, a consequence of multiple chronic life stressors (eg, racial discrimination) that engender increased exposure to interoceptive stress symptoms (eg, anxiety), which can ultimately lead to smoking as a means of immediate emotion regulation.

**Objective:**

This study aimed to culturally adapt and initially test a novel mobile intervention (ie, Mobile Anxiety Sensitivity Program for Smoking [MASP]) that targets anxiety sensitivity (AS; a proxy for difficulty and responsivity to interoceptive stress) among Black smokers. The MASP intervention is culturally informed to address interoceptive stress management difficulties among Black smokers and is thus hypothesized to facilitate smoking cessation.

**Methods:**

In phase 1, a total of 25 Black smokers with elevated AS will be administered MASP for 6 weeks. Following the completion of phase 1, we will further refine the MASP based on qualitative and quantitative data from participants to produce the final MASP iteration. In phase 2, a total of 200 Black smokers with elevated AS will be enrolled and randomly assigned to receive nicotine replacement therapy and either the smartphone-based National Cancer Institute QuitGuide app for standard mobile smoking cessation treatment or the MASP intervention. All participants in phases 1 and 2 will be enrolled remotely and will complete a web-based study screener; smartphone-based baseline assessment; daily smartphone-based ecological momentary assessments for 6 weeks; phone-based end-of-treatment qualitative interviews; and smartphone-based follow-up assessments at postbaseline weeks 1, 2 (quit date), 3, 4, 5, 6, 28, and 54 (weeks 28 and 54 follow-ups will be completed by phase 2 participants only). The MASP intervention is intended to offset barriers to treatment and encourage treatment engagement via smartphones.

**Results:**

This project was funded in September 2020. Phase 1 data collection began in January 2022. Phase 2 data collection is scheduled to begin in July 2022.

**Conclusions:**

If successful, data from this study will support culturally informed treatment approaches for Black smokers and, pending findings of efficacy, provide an evidence-based mobile intervention for smoking cessation that is ready for dissemination and implementation.

**Trial Registration:**

ClinicalTrials.gov NCT04838236; https://clinicaltrials.gov/ct2/show/NCT04838236

**International Registered Report Identifier (IRRID):**

DERR1-10.2196/38905

## Introduction

Black African American individuals are a tobacco-related health disparity group [1]. The prevalence of smoking among Black adults is 14.4% [2], exceeding that of non-Hispanic White individuals [2], regardless of socioeconomic status [3]. Although Black smokers smoke fewer cigarettes per day [4] and tend to begin smoking later in life than non-Hispanic White individuals [5], they evince greater levels of nicotine dependence [6] and serum cotinine [6-8] and are less likely to maintain abstinence despite more quit attempts [9]. Less quitting success may potentiate mortality rates for cardiovascular disease and other illnesses, which are overrepresented among this group [10]. A major contributing factor to smoking among Black adults appears to be their increased exposure to interoceptive stress symptoms, potentially stemming from and exacerbated by chronic stress related to systemic racism and racial discrimination [4,11-16].

Black adults are a health disparity group for interoceptive problems, including somatic symptoms, anxiety, stress, and pain [13-18], and evince stronger relations between negative emotional states and somatic experiences than White adults [19]. This is notable as Black adults diagnosed with anxiety disorders experience higher rates of hypertension, a condition for which Black adults are almost twice as likely to be diagnosed than White adults [20]. Black adults’ increased awareness of the negative outcomes of physical illnesses may further amplify somatic anxiety and interoceptive distress [21,22]. Anxiety sensitivity (AS) is among the most prominent cross-cultural constructs related to interoceptive distress. AS is a malleable, cognitive-affective factor that reflects the tendency to respond to interoceptive distress and anxiety [23,24]. AS is related to but distinct from negative affectivity and trait anxiety [24-30] and has demonstrated racial and ethnic, gender, age, and time invariance [27,29,31-33].

Smokers with elevated AS report greater tendencies to smoke to reduce negative affect, hold stronger beliefs that smoking will reduce negative affect, smoke in an inflexible manner in response to negative mood, perceive greater barriers to cessation, and experience increased expectations for adverse emotional distress during smoking deprivation [34-37]. The effects of AS on smoking seem to be independent of negative affect states such as anxiety or depression symptoms [38-40]. Smokers with high AS experience more intense nicotine withdrawal and craving during quitting, and higher levels of AS are related to greater odds of early lapse and relapse across clinical and nonclinical samples [41-43]. Withdrawal symptoms might be particularly salient in Black smokers with higher AS, as they may be more apt to perceive these internal sensations as uncontrollable because resources to regulate withdrawal symptoms (ie, adaptive-cognitive and behavioral skills) are likely diminished because of chronic stress exposure (eg, microaggressions, racism, and stress-related burden because of racial discrimination) [44,45]. In turn, Black adults with higher AS may be motivated to smoke to reduce emotional and interoceptive distress.

AS reduction has been integrated into in-person substance use treatment programs among largely non-Black samples [46]. Such programs, originally developed by our team [47,48] and now replicated by other researchers [49-52], have found that reducing AS via cognitive behavioral therapies before quitting increases the odds of smoking cessation [53,54]. Although such effects tend to be the most robust among smokers with higher AS [55], reduction of AS, even among smokers with moderate levels of AS, is helpful in reducing tobacco-related stressors such as withdrawal [55] and improving mental health [56]. However, all previous AS reduction work for smoking has thus far been conducted via in-person approaches within non-Black samples, except for our successful small-scale pilot study, which included a large proportion of Black smokers (66.7%) [57]. Given that smoking can produce perceived or objective acute anxiolytic effects [58], Black smokers with high AS may be more likely to return to smoking following a quit attempt, in part, to alleviate abstinence-induced increases in anxiety, underscoring the need for a culturally tailored intervention to address internal agitation in the context of smoking cessation treatment.

To date, no smoking cessation treatments for Black smokers have focused on sensitivity to interoceptive stress despite the importance of somatic symptoms, including anxiety, stress, and pain, among this population. Furthermore, previous efforts to evaluate the effectiveness of culturally targeted smoking cessation programs for Black smokers have mainly used minimal intervention strategies and group counseling and were nonrandomized trials [59]. We developed and preliminarily tested our novel, integrated, smartphone-delivered intervention for interoceptive stress and smoking (ie, Mobile Anxiety Sensitivity Program for Smoking [MASP]) using a single-arm pilot trial design [57]. This intervention is based on theory and empirical evidence of the importance of interoceptive stress for smokers [60]. The purpose of our initial pilot of the MASP 1.0 (N=12; mean age 45 years; 66.7% Black) was to assess app engagement and determine whether there was an initial signal of app impact on cessation. Participants self-initiated interoceptive exposure exercises 6 times on average, self-initiated *relaxation exercises* 6 times on average, and accessed *treatment audio files* 2 times on average [57]. Biochemically verified abstinence was observed in 25% of the participants at the 6-week follow-up visit (4 weeks after quitting), which are striking and clinically important, given that (1) this was only the initial version of the treatment (MASP 1.0), and (2) smoking cessation success rates for Black smokers are typically low.

The objectives of this National Institute on Minority Health and Health Disparities–funded trial (U54MD015946) are to refine and evaluate the feasibility, acceptability, and efficacy of a novel, culturally tailored, smartphone-delivered MASP app for Black smokers. In phase 1, MASP 1.0 was refined and culturally adapted based on theoretical and empirical guidelines [61] and the initial participant feedback collected during the MASP 1.0 pilot to produce the MASP 2.0. Black smokers with elevated AS (N=25) will use the MASP 2.0 for 6 weeks (ie, the intervention period). Following the intervention period, participants will complete a qualitative interview, which, along with quantitative data and feedback from the community research advisory board as part of the Community Engagement Core of the health institute, will be analyzed to guide intervention refinement to produce MASP 3.0. In phase 2, we will conduct a randomized controlled trial (RCT) of 200 Black smokers with elevated AS who will be randomly assigned to either the MASP 3.0 or the smartphone-based National Cancer Institute (NCI) QuitGuide app, which is considered the smartphone-based standard of care for smoking cessation [62,63]. Mechanisms underlying the intervention effects, including the primary mechanism of AS and secondary mechanisms (ie, anxiety and depression symptoms, stress-based burden of racial or ethnic discrimination, and nicotine withdrawal or craving), will be evaluated. The indirect effects of the MASP intervention (vs QuitGuide) on smoking cessation via these mechanisms of change will be evaluated. Finally, we will explore whether stress associated with perceived racial discrimination, acculturation, ethnic identity, and COVID-19 may function as moderators of smoking outcomes.

## Methods

### Ethics Approval

The University of Houston (reference number: 12747) and the University of Oklahoma Health Sciences Center (reference number: STUDY00000360) institutional review boards approved the protocol presented in this study. This trial was registered at ClinicalTrials.gov (NCT04838236).

### Study Eligibility

Adults aged ≥18 years are eligible to participate in this study if they self-identify as Black and report high AS (ie, Short Scale Anxiety Sensitivity Index [64] score of ≥5), daily smoking (minimum of 10 cigarettes per day) for at least 2 years, motivation to quit smoking (>5 on a 10-point scale) [65], willingness to complete all study surveys and assessments, willingness to use nicotine replacement medications (nicotine replacement therapy [NRT]; nicotine patch and lozenges), and desire to quit smoking 2 weeks after completion of the baseline survey and receipt of study materials. The exclusion criteria include current or intended participation in a concurrent substance use treatment program, ongoing psychotherapy of any duration directed specifically toward the treatment of anxiety or depression, not being fluent in English, current use of any pharmacotherapy or psychotherapy for smoking cessation not provided by this study, a legal status that would interfere with participation, cognitive impairment (assessed via the 6-item Cognitive Impairment Test [66]), being non-Black, and being aged <18 years. Those who participated in the previous phase of the study are not eligible to participate in future study stages.

### Recruitment and Procedure

Participant enrollment in the study and all study procedures will be conducted remotely. We will advertise through community organizations that promote smoking cessation initiatives, social media, and internet outlets. Study screening will be completed via REDCap (Research Electronic Data Capture; Vanderbilt University) when a participant clicks on the study advertisement. Individuals who prescreen as eligible via the REDCap assessment will be phoned to provide informed consent and complete the final eligibility screening for the study. To reduce the likelihood of fraudulent enrollments in the study, potential participants will be required to text a picture of their pack of cigarettes in real time as evidence that they are smokers, and they will be required to text a picture of their ID or a piece of mail with their name and address on it. Persons who will be deemed eligible and willing to participate during the enrollment phone call will be provided with information about the purpose, goals, and procedures of the study and asked to download the Insight app (University of Oklahoma Health Sciences Center) onto their personal smartphone or mailed a smartphone if they do not have one that is compatible with the Insight platform.

**Figure 1:**
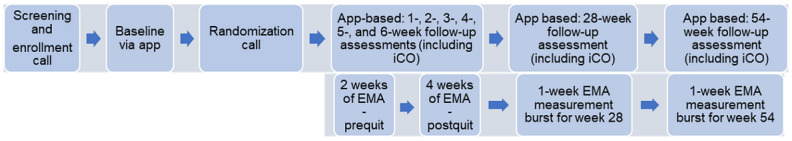
Participant flow. EMA: ecological momentary assessment.

Once the Insight app is downloaded, participants will be given a unique single-use code that will enable them to use the app to complete the baseline assessment on their personal or study-provided smartphone. After the baseline assessment is completed, participants will be mailed a Bedfont iCOquit smokerlyzer, which reports levels of carbon monoxide (CO) in expired breath to verify self-reported smoking status (phases 1 and 2). Upon receipt of the iCO, participants will complete a brief phone call with the study staff to review all app functions and be oriented to the iCO. In phase 2, REDCap will be used to randomize participants to the MASP 3.0 or QuitGuide during this brief call. Participants will then use the assigned intervention via the app for 6 weeks and complete daily ecological momentary assessments (EMAs) during the intervention period. App-based follow-ups will occur 1, 2, 3, 4, 5, and 6 weeks after the baseline for all participants; phase 2 participants will also complete follow-ups at 28 and 54 weeks after the baseline. All interactions with the QuitGuide and MASP apps will be date- and time-stamped, and the app feature use will be recorded for future analysis. All daily surveys and follow-up assessments (both treatment arms) will be completed using the Insight app, and a phone-based qualitative interview will be completed at the end of the 6-week intervention period. We have developed a system of procedures that includes contacting participants to encourage retention (ie, calling, texting, and emailing participants who have not completed follow-up assessments). Participants who will borrow smartphones will be asked to return them after the final follow-up assessment. In addition to randomization in phase 2, similar procedures will be followed in phases 1 and 2 (Figure 1).

Following the completion of the smartphone-based baseline assessment in phase 2, we will block randomize participants by binary sex (assigned at birth) and baseline smoking level (rate per day) to ensure that the cell sizes are largely equivalent for maximally useful subgroup testing [67]. Variable-sized permuted block randomization (block sizes will vary from 6 to 10) will be used. Before data analysis, the balance of randomization will be checked and controlled for imbalanced factors.

### Study Conditions

#### Both Conditions

**Figure 2:**
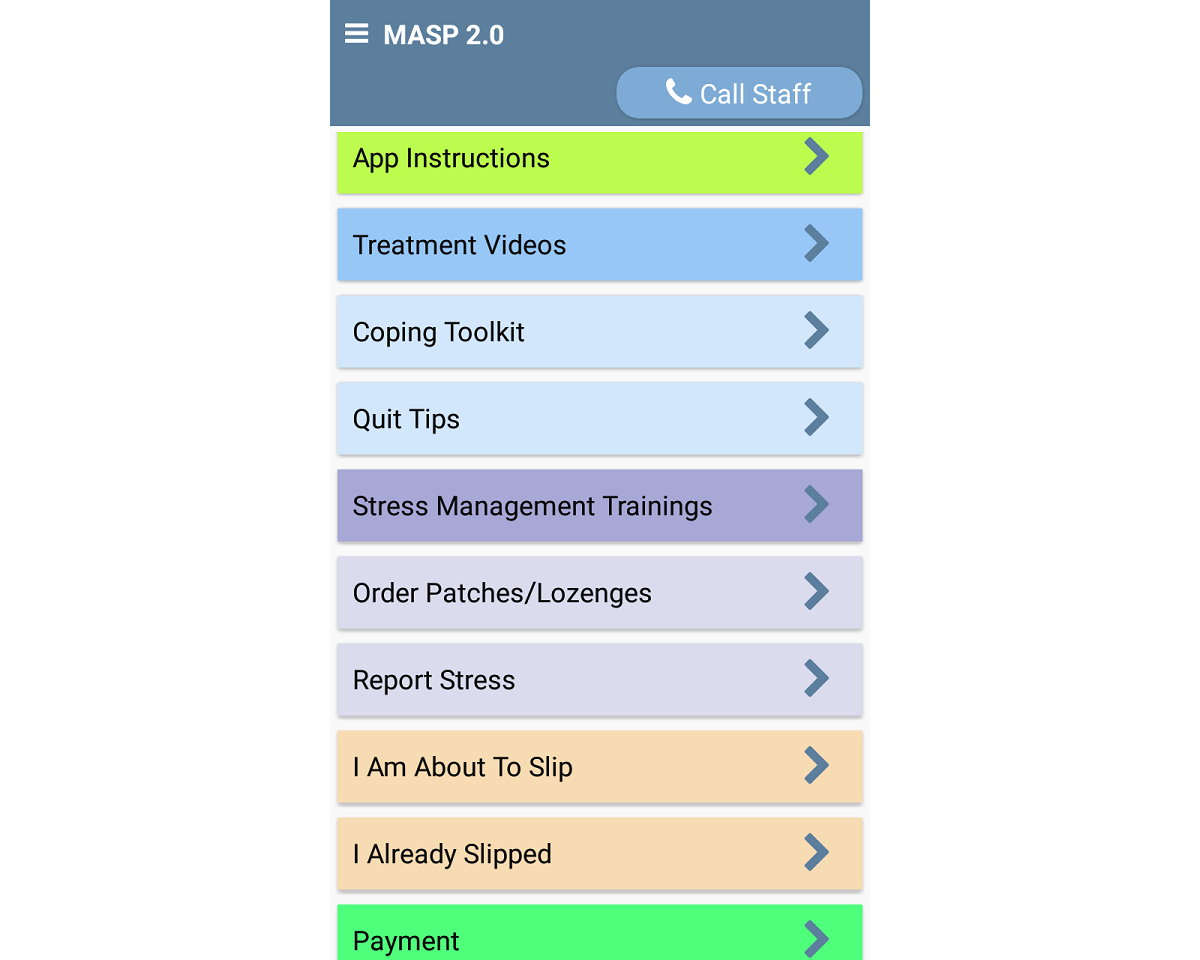
The Mobile Anxiety Sensitivity Program for Smoking app.

Clinical guideline recommendations indicate that all smokers attempting to quit should receive pharmacotherapy [68]. Thus, the MASP (phases 1 and 2) and QuitGuide (phase 2) participants will receive a combination of NRT (transdermal nicotine patch and nicotine lozenges) during the first 4 weeks after quitting. Additional NRT (up to 8 additional weeks) may be requested beyond the first 4 weeks by clicking a button on the app home screen or clicking the *Call Staff* button on the app home screen (Figure 2). Note that 66% of those enrolled in one of our previous studies used a similar app button and feature to order the NRT [62]. We chose the patch and lozenges because of the extensive empirical literature supporting their effectiveness and safety, ease of use, and relatively benign side effect profile, as well as the increased efficacy of combination NRT [69,70]. The MASP app will send an encrypted email to alert the treatment team of NRT requests, and participants will be mailed an additional NRT.

#### NCI QuitGuide Condition

The NCI QuitGuide app is a free smartphone app available through Smokefree.gov. QuitGuide is one of the few apps that are consistent with many of the recommendations of the Clinical Practice Guidelines for quitting smoking [68]. Individual-level app feature use is accessible by request from Smokefree.gov server. The QuitGuide app aims to help smokers understand their smoking patterns and develop the skills needed to quit smoking. Participants who are randomly assigned to the QuitGuide condition will use their personal smartphone or receive a study smartphone if they do not have one, which will be preloaded with the QuitGuide app and preset for a quit date 2 weeks after randomization into an intervention group. Participants will receive information on how to use the QuitGuide app features during the randomization phone call. QuitGuide participants will also download an Insight EMA app stripped of all the MASP intervention features. Table 1 for a list of QuitGuide features.

**Table 1:** Comparison of phase 2 treatment conditions.

App components			MASP^a^		QuitGuide
EMA^b^			✓		✓ (add-on for this study)
Set a quit date			Quit date set to 2 weeks after randomization and receipt of the iCO via mail		Quit date set to 2 weeks after randomization and receipt of the iCO via mail
Share quit information on social media					✓
Smoking cessation psychoeducation			✓		✓
Content specific to Black smokers			✓		
NRT^c^ tips and use advice			✓		
AS^d^ psychoeducation			✓		
**On-demand tips and exercises**					
	Coping with cravings	✓		✓	
	Coping with mood	✓		✓	
	Coping with stress	✓			
	Inspirational messages	✓		✓	
Scheduled tips					✓
Treatment messages tailored to currently present smoking lapse triggers			✓		
**Coping toolkit**			✓		
	Guided relaxation and mindfulness exercises	✓			
	Challenging automatic thoughts	✓			
	Tips for coping with stress	✓			
	Interoceptive exposure	✓			
	Resources to help distract participants if they experience elevated distress	✓			
Individualized quit plan					✓
Document smoking triggers					✓
List reasons for quitting					✓
Saving from smoking fewer cigarettes					✓
Creating journal entries					✓

^a^MASP: Mobile Anxiety Sensitivity Program.

^b^EMA: ecological momentary assessment.

^c^NRT: nicotine replacement therapy.

^d^AS: anxiety sensitivity.

#### MASP Condition

##### Overview

The updated MASP 2.0 app focuses on general interoceptive stress-related issues and interoceptive stress that may be specific to Black smokers [71]. The MASP 2.0 integrates a standard cognitive behavioral therapy smoking cessation protocol (clinical practice guidelines) [68] with transdiagnostic treatment for AS reduction in a culturally adapted framework. Specifically, the MASP 2.0 provides (1) standard treatment on a schedule, (2) scheduled and cued interoceptive exposure sessions, (3) personalized and tailored messaging after each EMA (before and after quitting), and (4) *on-demand* features (eg, coping toolkit and cognitive restructuring exercises) before and after quitting. Culturally specific elements (eg, education about menthol tobacco products, history of tobacco marketing to Black smokers, concerns about pharmacotherapy, religion and spirituality coping, and the impact of racism and racial discrimination on stress and smoking) are included throughout the MASP 2.0.

##### Treatment on a Schedule

The MASP 2.0 includes 16 intervention videos of 4 to 6 minutes (adapted from traditional AS reduction treatments [72] and the MASP 1.0 audio files), which are delivered through the MASP 2.0 app over the 6-week intervention period. Notably, Black voice actors (men and women) will present all intervention content. New videos will be offered at the completion of the prequit morning and evening EMAs. Participants will have the option to watch videos immediately or later by clicking the on-demand *Treatment Videos* button (Figure 2). Videos can be watched as many times as desired, and the app records the date, time and location when each video is watched (both initiation and completion).

The brief videos provide psychoeducation on multiple topics, including the relationship between stress and smoking, managing uncomfortable sensations, unhelpful thinking, smoking as a temporary coping mechanism to avoid experiencing negative emotions, thinking flexibly, myths about smoking, coping with others smoking nearby, nicotine withdrawal, the importance of using NRT, stress management, interoceptive exposure techniques, and strategies for cessation and relapse prevention. Culturally tailored content (eg, tobacco industry marketing of menthol cigarettes to Black communities, effects of discrimination on smoking and relapse, concerns about pharmacotherapy, chronic stress and race, and the role of AS in interoceptive stress in Black adults) is infused throughout the brief videos.

##### Exposure Sessions

Internet-based interoceptive exposure is well-tolerated, acceptable, and effective [73,74]. To target AS, graduated exposure to anxiety- and distress-provoking situations and response prevention is introduced in the MASP videos. These exposure exercises were created for the MASP 1.0 study (ie, overbreathing, straw breathing, running in place, chair spinning, and head rush) [57]. The app reminds participants of the importance of completing the exposure sessions 3 times per day during random EMAs. Participants will be instructed to click the *Stress Management Trainings* button to initiate an interoceptive exposure session (Figure 2). The MASP app randomly selects 1 of the 5 exercises each time the *Stress Management Trainings* button is pressed. The participants will be guided through interoceptive exposures by explaining the purpose of the activity and how to perform it, normalizing the physical symptoms experienced during the exercise and relating this experience to the process of quitting smoking (eg, tolerance of physical symptoms related to anxiety, discrimination, and withdrawal). As in the MASP 1.0 pilot [57], when the participant is ready to begin, the app assesses the level of distress (0-100 scale), shows a countdown timer while the exercise is being completed, and assesses the level of distress again (0-100 scale) once the countdown expires. The app suggests repeating the exercise up to 3 additional times if the current reported distress is >50 on a 1 to 100 scale. This strategy aims to increase habituation to feared sensations. In the MASP pilot study [57], participants accessed the stress management training exercises on 6 of the 13 days that preceded their quit date.

##### EMAs With Tailored Real-time Treatment Messages

During the 2-week prequit period, a message will be delivered at the end of each EMA (5 per day), which will focus on increasing motivation and provide information about cessation (eg, “Everyone experiences negative emotions, such as stress. These emotions do not last forever, but can lead to relapse. Make a specific plan to cope with such feelings”). During the 4-week postquit intervention period, participants will receive personally tailored messages at the end of each EMA based on their responses to EMA items and their reported likelihood of smoking today (ie, 0%-100%). The type of message (eg, motivational, coping with urges or stress, or coping with other smokers nearby) that is delivered at the end of each EMA is recorded in the database; therefore, we may analyze the effect of messages on targeted smoking lapse triggers and anxiety or depression in future EMAs. Participants will receive general quitting advice when they report a 0% chance of smoking that day during EMAs. In addition, the participants will be instructed to review and practice interoceptive exposure exercises to further normalize the symptoms of anxiety and withdrawal to prevent lapse.

##### On-Demand Features

**Figure 3:**
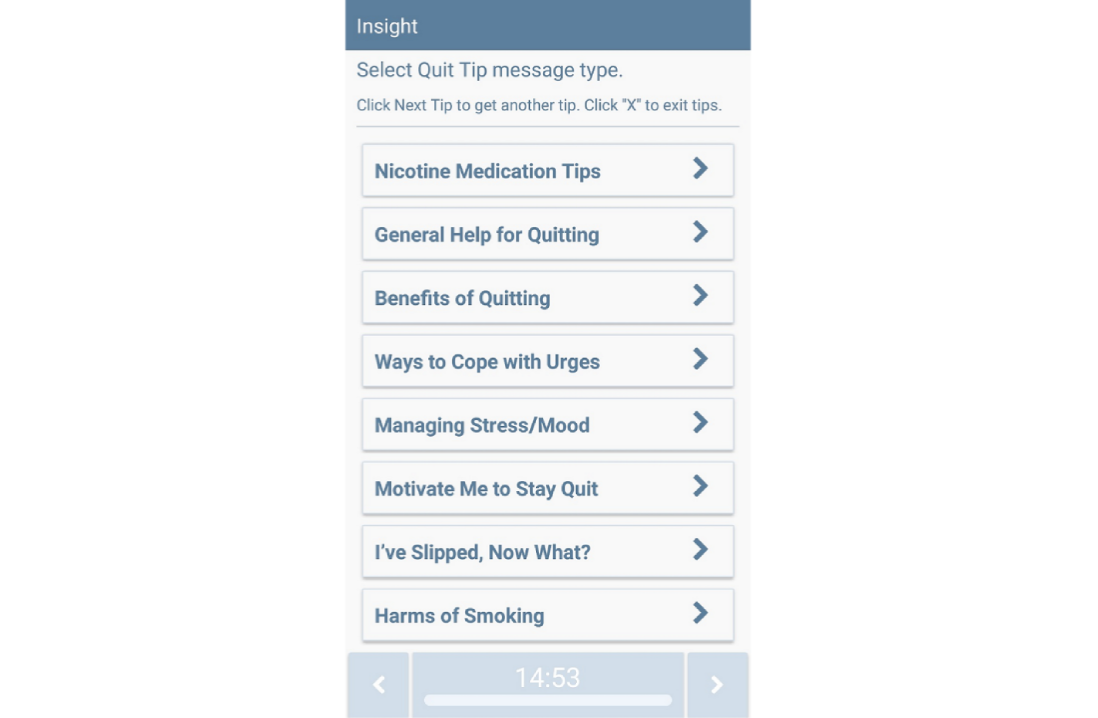
The Mobile Anxiety Sensitivity Program for Smoking Quit Tips feature.

**Figure 4:**
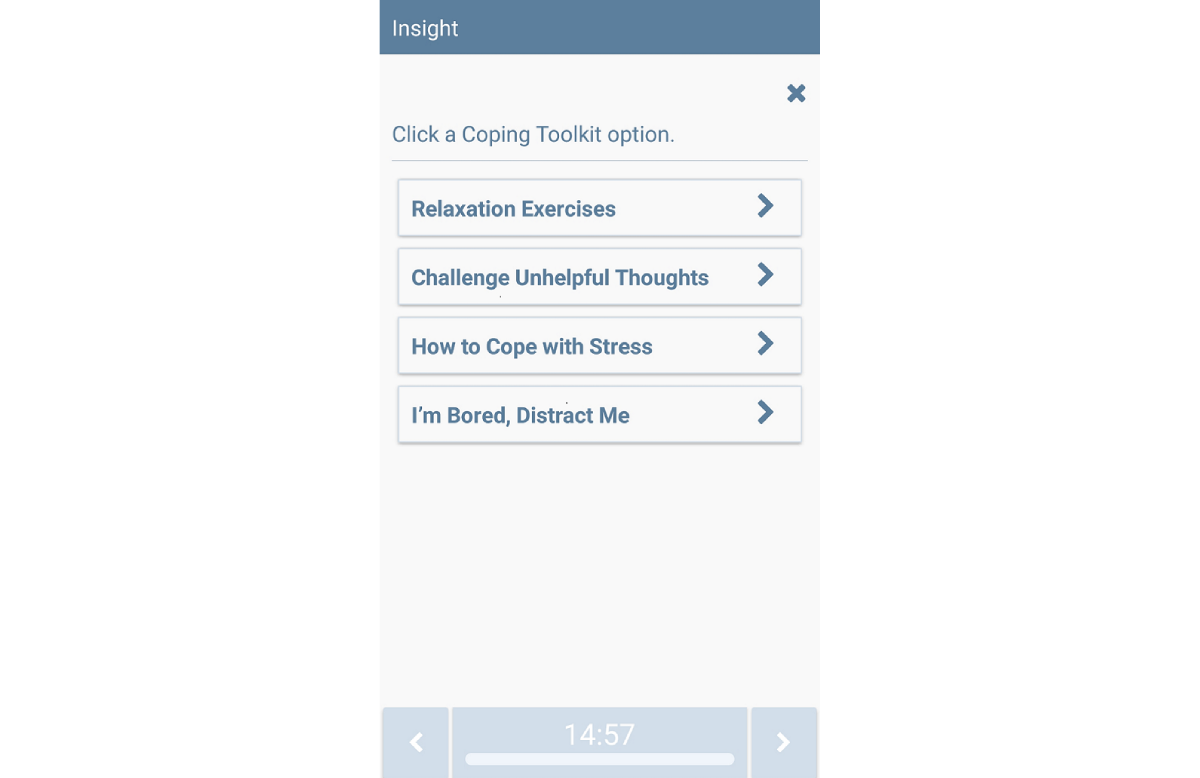
The Mobile Anxiety Sensitivity Program for Smoking Coping Toolkit feature.

On-demand tips and relaxation exercises will be available through buttons on the MASP home screen. The research team developed hundreds of unique messages for Smart-T studies [62,75] to address various lapse risk triggers and reduce repetition. MSB, LG, and MJZ led the modification of Smart-T messages and the development of new, short, tailored treatment messages for this study. Participants will be instructed to click the on-demand *Quit Tips* feature when they want to learn more about NRT or coping with smoking lapse triggers (see Figure 3 for the menu of MASP *Quit Tips*); click the *Coping Toolkit* button when they want to access relaxation or mindfulness videos, app-guided exercises focused on challenging automatic thoughts, tips on ways of coping with stress; and click a *Distract Me* button that links to funny or cute videos (Figure 4 for MASP *Coping Toolkit* options). Each time the *Coping Toolkit* option is pressed, a new message or video is presented.

##### MASP 3.0 Development

We will assess the perceived utility of the MASP 2.0 using the standardized System Usability Scale (a participant-completed, reliable, and valid metric for measuring usability and acceptability of technologies) [76-78] and phase 1 qualitative interviews conducted via phone at the end of treatment (week 6). The semistructured qualitative interviews will be conducted by a trained research assistant and audio recorded. These assessments will focus on the ease of interacting with the app, usefulness of app features, how the app can be improved, how sociocultural factors that affect Black smokers could be further woven into the intervention, and the willingness to refer the app to a friend. The refinement process will yield the MASP 3.0. We anticipate that the MASP 3.0 will retain all the key elements of the MASP 2.0 but include improved content that is more culturally tailored based on participant feedback.

### Measures

#### Overview

Baseline and follow-up assessment data will be primarily collected using smartphones through the Insight mobile health (mHealth) platform app software [79]. This approach will reduce data entry errors and the need to retain paper copies of raw data. Each question will appear on the smartphone screen, and the participant will respond by touching their answer on the touch screen. The amount of time needed to complete the questionnaires will vary. The baseline assessment requires up to 30 minutes to complete, and follow-up assessments require up to 15 minutes to complete for both study phases.

#### Screening

The initial web-based REDCap screening will be completed when the participants click an advertisement for the study. The screener includes an assessment of demographics (eg, sex, age, and race and ethnicity), smoking history, motivation to quit, smartphone and data plan details, and other eligibility criteria. Those that prescreen as eligible via the REDCap screener will be invited to complete a phone call, wherein additional inclusion and exclusion criteria will be assessed. The Rapid Estimate of Adult Literacy in Medicine—Short Form [80] will be used to assess literacy (an English literacy level higher than sixth grade is required to complete the EMAs). Socioeconomic status will be measured as income and the highest level of education. The 6-item Cognitive Impairment Test will be used to assess significant impairment in cognitive function (participants with scores <8 will be excluded) [66]. The Short Scale Anxiety Sensitivity Index is a 5-item measure that will be used to assess AS [64].

#### Baseline Assessment

The baseline assessment (completed via Insight) includes demographic questions (eg, employment status, income, and insurance), subjective social status [81], Black racial identity [82], acculturation [83], discrimination [84,85], COVID-19 [86], coping [87], attendance at religious services [88], self-rated health [89], health-related quality of life [90], heaviness of smoking [91], tobacco use history, readiness to quit smoking [92], self-efficacy for quitting smoking [93], AS [64], perceived stress [94], chronic pain [95], mindfulness [96], anxiety [97,98], depression [99], social support [100], sleep [101], alcohol use [102], marijuana use [103], and other health behaviors [104].

#### Follow-up Assessments

Follow-up assessments will be completed using the Insight app at weeks 1, 2 (quit date), 3, 4, 5, 6, 28, and 54 (52 weeks after quitting). Follow-up surveys will be distinguishable from EMAs (ie, participants will self-initiate the assessment by clicking a button labeled *Follow-up Survey* and receive separate compensation for completing these assessments). Importantly, participants will also be asked to complete a phone-based qualitative interview with the study staff at the 6-week follow-up (phase 1 and phase 2). If a participant does not complete the brief app-based follow-up assessments, they will be contacted via call, text, and email to remind them to complete them.

#### Smoking Outcomes

Biochemically confirmed smoking status will be assessed 3 times per week using the Insight platform (phases 1 and 2). Consistent with most published smoking cessation RCTs (see the Clinical Practice Guidelines) [68] and best practices (see the study by Benowitz et al [105]), our primary study outcome is the biochemically confirmed, 7-day, point prevalence abstinence (PPA) 4 weeks (phase 1) or 52 weeks (phase 2) after the scheduled quit date. Secondary outcomes (eg, time to first lapse and longest time of quitting) will also be examined. Smoking abstinence will be assessed daily via self-report (ie, EMA; weeks 1-6, 28, and 54 [phase 2 only]). The low-cost Bedfont iCOquit smokerlyzer will be used to verify smoking status during the follow-up assessments (ie, quit date; weeks 1-4, 26, and 52 after quitting). Participants will be prompted by the Insight app to connect the iCO device to the smartphone (simply by pushing the power button) and follow step-by-step directions to complete the iCO test. The results of these tests will be automatically date- and time-stamped and saved to our server. We have integrated automated and secure (ie, encrypted) facial recognition software to ensure that only the participants complete the prompted iCO tests. Our CO criteria for abstinence is consistent with numerous studies using cutoffs <7 ppm [106-112]. The half-life of CO is up to 8 hours, depending on a variety of factors (eg, time of day and daily smoking rate) [113]. Expired CO is a valid indicator of smoking status and cessation outcomes and compares favorably with cotinine and other biochemical measures that have longer detection windows [114-116].

#### EMA and Types

##### Overview

EMA is currently the most accurate way of measuring phenomena in real time in natural settings [117,118]. EMA items identify fluctuations in key variables that predict study outcomes with less bias than traditional in-person assessments. EMA data will be used to identify moments of high smoking lapse risk, tailor MASP treatment, and identify the treatment mechanisms.

The EMA methodology used in this study is similar to that used in our previous studies and by other researchers [57,62,75,119-126]. EMA items assess multiple constructs hypothesized to be related to smoking lapse. A total of 3 types of EMAs will be used: daily diaries, random sampling, and event sampling. Random and daily diary EMAs will be prompted and initiated by phone. The phone will audibly and visually cue these EMAs for 30 seconds. If a participant does not respond after 5 prompts, the assessment will be recorded as *missed*. All assessments will be date- and time-stamped for future analyses.

##### Daily Diary

Daily diary EMAs in the morning will be completed 30 minutes after waking, and daily diaries in the evening will be completed 75 minutes before bedtime. Questions will ask about the previous day and current thoughts, feelings, and behaviors (eg, “How many cigarettes did you smoke yesterday?” and “How many standard drinks of alcohol did you have today?”). Participants will also be asked about smoking cessation medication use and sleep quality for the previous day. Finally, as has been done in our previous work, participants will be prompted to blow into the Bedfont iCO 3 times per week during the evening daily diaries to confirm their self-reported smoking status.

##### Random Sampling

Participants will be prompted to complete random EMAs scheduled to occur during each participant’s normal waking hours 3 times each day (6 weeks total for phase 1 and 8 weeks total for phase 2). Participants will rate their affect by indicating the extent to which they agree or disagree with statements (using a 5-point scale from strongly disagree to strongly agree; eg, “I feel irritable, restless, stressed, alert”). In addition, participants will be asked about current smoking triggers (eg, “I have an urge to smoke” and “I am motivated to AVOID smoking”) and indicate current depression (eg, “Rate your current level of depression [feeling sad]”) and anxiety (eg, “Rate your current level of anxiety [feeling nervous]”). Participants also will describe their current environment (eg, home and work) and social settings. The other relevant constructs will be assessed during random sampling.

##### Event Sampling

Participants will be asked to self-initiate smoking assessments (prequit period), lapse assessments (postquit period), and stress assessments (pre and postquit periods) as follows:

1. Smoking Assessments: During the prequit period, participants will be instructed to click a *Record Cigarette* button each time they smoke. Approximately 10% of the time, participants will be asked to answer questions about their affect, stress, and experiences while smoking (eg, “Smoking was pleasurable” and “Smoking improved my mood”). All smoking assessments will be date-, time-, and location-stamped for future analysis.
2. Lapse Assessments: Participants will be instructed to complete lapse assessments (by clicking the *I Am About to Slip or I Already Slipped* buttons) each time they smoke after their quitting date. The lapse assessment items will be nearly identical to those presented in the random and urge assessments. However, questions will be worded to separately assess the participants’ responses immediately before and after the lapse. Postlapse assessments will also query about the reinforcing value of the lapse cigarette or cigarettes and the causes of the lapse.
3. Stress Assessments: Participants will be instructed to complete *Report Stress* assessments each time they experience *a significant increase in stress*, and answers to survey questions should be focused on immediate thoughts or feelings. Importantly, MASP prequit EMAs will be followed by predetermined treatment messages, and MASP postquit EMAs will be followed by treatment messages tailored to the participant’s responses and current situation. Those assigned to the QuitGuide group will complete EMAs that are identical to the MASP group but will not receive tailored intervention messages.

##### EMA Alert Settings

During the enrollment call, a phone setup wizard will be used to set participant sleep and wake times for each day of the week (sleep and wake times can be changed for those with variable schedules). This practice reduces the likelihood that the phone will ring when participants are sleeping. In addition, participants may delay EMAs by up to 30 minutes by clicking on the snooze assessment option when an EMA is prompted.

##### Data Loss Prevention

To overcome the potential loss of data if participants lose their phones (<1% of phones have been lost in most studies), the phones will be programmed to connect to our secure server multiple times each day to upload encrypted data. This will ensure that very little of the collected EMA data are lost. This tactic also allows researchers to remotely monitor each participant’s EMA completion rate and call participants when the rate is low. Importantly, EMA data are password protected and encrypted on a study phone. Thus, the study data are only accessible to the research team. If a phone is lost, it will be remotely wiped, and only one replacement phone will be provided to each participant.

### The Insight Platform

#### Overview

The mHealth Shared Resource at the University of Oklahoma Health Sciences Center and Stephenson Cancer Center have developed the Insight mHealth platform, which offers resources that empower researchers to build, test, and launch technology-based assessment and intervention tools [79]. The mHealth resource uses a program manager, 4 project coordinators, and 4.5 computer scientists and engineers who develop and maintain web applications, mobile apps, and relational databases. The applications are developed using state-of-the-art cross-platform design tools.

#### Smartphone Training

We have developed and repeatedly implemented a brief, user-friendly training protocol for those with limited smartphone experience. Participants will receive training on how to use all features of the MASP app (phases 1 and 2), the EMA-only Insight app (phase 2), and the QuitGuide app (phase 2) during their enrollment phone call and will have access to an *App Instructions* button in the app to remind them about how each app feature functions (Figure 2). We achieved high EMA compliance rates (eg, 82%-87% of all EMAs completed) using similar protocols in samples of socioeconomically disadvantaged and nondisadvantaged adults [62,75,126,127]. The smartphones will automatically collect intervention delivery data (ie, number of minutes treatment videos are watched and number of times features are used).

### Compensation

All participants that enroll in the study will receive a Greenphire Mastercard gift card to facilitate payment for completing study surveys. Greenphire offers an auditable mechanism for all study payments. Participants will receive US $30 for completing the baseline assessment and US $15 for completing follow-up assessments at 1, 2 (quit date), 3, 4, and 5 weeks after the baseline. Participants will receive US $45 for completing the 6-week follow-up, which includes an app-based survey, iCO, and phone-based qualitative interviews. Furthermore, phase 2 participants will receive US $50 for completing the 28- and 54-week follow-up assessments (via Insight) and iCO breath tests. In addition, eligible participants will be compensated for completing the EMAs during their study participation (ie, weeks 1-6, 28, and 54). Specifically, phase 1 and phase 2 participants who complete 50% to 74% of the brief EMAs (5 per day × 7 days=35 weekly EMAs) will receive US $60 or US $80 for 6 or 8 weeks of EMA, those who complete 75% to 89% of assessments will receive US $120 or US $160 for 6 or 8 weeks of EMA, and those who complete ≥90% of their EMAs will receive US $150 or US $200 for 6 or 8 weeks of EMA. Participants can click the *Payment* button on the app home screen at any time for an up-to-the-moment summary of the presented EMAs and current completion rate. Payments for completing EMAs will be loaded onto Greenphire cards following weeks 6 (phases 1 and 2), 26 (phase 2), and 54 (phase 2). Participants will not be compensated for accessing the on-demand app features or for competing treatment components.

### Statistical Analyses

Qualitative and quantitative data will be collected in phase 2 and will be used to refine the MASP 2.0 into the MASP 3.0 for testing in phase 2. The feasibility and utility of the MASP 2.0 app will be examined by quantifying the use of the MASP 2.0 features (eg, number of assigned videos that are watched) and evaluating participant opinions about the usefulness and helpfulness of the MASP 2.0 features (eg, automated treatment messages that follow EMAs, interoceptive exposure sessions, and treatment videos). During the end-of-treatment qualitative interviews, we will elicit information on what participants liked about the MASP and QuitGuide apps and how the apps could be improved and identify barriers to app engagement. Semistructured qualitative interviews will be audio recorded and transcribed. A team-based approach will be used to determine the appropriateness of incorporating the suggested changes into the refined phase 2 version of the MASP (MASP 3.0). This approach is consistent with the systematic and reflexive interviewing and reporting methods [128] and will help systematically organize qualitative data to guide the refinement of the MASP 2.0 following phase 1 and before testing in phase 2. In addition, a similar procedure will be followed to organize and address qualitative and quantitative data collected as part of the second phase of the study.

Multiple approaches to modeling changes in abstinence will be used to evaluate our primary hypothesis that the MASP 3.0 will result in higher rates of smoking abstinence relative to the NCI QuitGuide app. We will first use the biochemically verified measure of 7-day PPA, which will be collected via Bedfont iCO at each major assessment, as the primary indicator of abstinence. PPA will be defined as no smoking, not even a puff, in the 7 days before any assessment. We will estimate between-group differences by calculating the odds ratio effect sizes (with 95% CIs) for PPA at each follow-up assessment. We will then conduct a series of conditional latent growth models to examine the impact of treatment conditions on abstinence trajectories. We will use multilevel structural equation models to examine the effects of the MASP 3.0 intervention on the longitudinal course of smoking behaviors, as measured by daily dairy and random sampling EMA data. We will conduct survival analyses using Cox proportional hazard models to assess time to lapse and time to relapse and whether the treatment condition predicts patterns of smoking in survival analysis. Similar modeling procedures will be used to determine the impact of treatment conditions on AS, anxiety symptoms, depression symptoms, stress burden, nicotine withdrawal, and craving and whether improvements in smoking cessation outcomes are mediated by reductions in AS and secondary mechanisms.

## Results

The phase 1 smartphone app has been developed (Figures 2-4), and data collection began in January 2022. Phase 2 data collection is expected to begin in July 2022.

## Discussion

### Principal Findings

This study is the first to culturally tailor a smoking cessation app for Black smokers and the first to incorporate AS intervention components into an automated smoking cessation intervention. We hypothesize that participants will use the MASP app features, report that the intervention is useful and helpful, and report that they would refer a friend to use the app. In addition, we hypothesize that participants randomized to the MASP 3.0 intervention will have greater smoking cessation rates 52 weeks after the scheduled quit date than those assigned to the QuitGuide intervention. Furthermore, this study will advance the understanding of the mechanisms related to smoking relapse. Specifically, we will examine the relationships among self-reported anxiety, depression, avoidance, craving, and withdrawal symptoms as they relate to AS and smoking behavior. A deeper understanding of the mechanisms related to quitting success among Black smokers with AS will improve future interventions by isolating specific targets for the timing and content of intervention messages.

Smartphone interventions have great potential to provide low-cost and scalable treatments for diverse populations. In 2021, overall, 85% of US adults reported owning a smartphone, and ownership is high among minoritized populations (83% among Black adults), as well as individuals with low socioeconomic status (76% among those earning <US $30,000 per year) [129]. The dynamic nature of AS and related symptoms (eg, anxiety, depression, and withdrawal or craving) makes a mobile intervention ideally suited to addressing risk factors and characteristics that vary from person to person and over time. By providing an automated intervention that tailors treatment content based on psychological and environmental contexts in real time, this study has the potential to provide precision treatment that cannot otherwise be obtained via traditional, in-person therapy. If effective, this type of automated, scalable, and culturally informed smoking cessation app can be easily incorporated into other *real-world* settings to reduce health disparities.

### Future Work

Studies are needed to translate and culturally adapt effective in-person cessation interventions into mobile, remotely delivered treatments that have greater cessation and reach potential for historically oppressed and underserved populations, such as Black smokers. Given that past work supports the feasibility of providing smoking cessation care via mobile technology [57,62,63], integrated, mobile AS smoking treatment represents a critical *next step* to addressing tobacco-related health disparities among Black smokers. This study is designed to extend our past AS smoking work and the larger field of smoking–emotional disorder comorbidity by refining (phase 1) and testing a fully automated, culturally tailored, mobile AS smoking cessation intervention for Black smokers in an RCT (phase 2). On the basis of the mechanisms of action observed in this study, future work will focus on refining the intervention to more effectively target key mediators between AS and quitting success. Pending efficacy findings, the MASP intervention will be poised for national dissemination and implementation across health care settings.

## Appendix

Multimedia Appendix 1 Peer review summary statement from the National Institute on Minority Health and Health Disparities Special Emphasis Panel NIMHD Research Centers in Minority Institutions (RCMI) (National Institutes of Health, USA).

## Abbreviations

AS: anxiety sensitivity
CO: carbon monoxide
EMA: ecological momentary assessment
MASP: Mobile Anxiety Sensitivity Program for Smoking
mhealth: mobile health
NCI: National Cancer Institute
NRT: nicotine replacement therapy
PPA: point prevalence abstinenceRCT: randomized controlled trial
REDCap: Research Electronic Data Capture

## Data Availability

The authors will provide deidentified data from this project to interested individuals 1 year after achieving the aims of the project (ie, publication of the main outcome paper). These data will be provided in a digital format with clear labels for all variables. Data will be released directly by the investigators, providing evidence for their institution’s institutional review board approval for planned analyses of the data. The authors’ team will be available to address any queries.
