# Supplementary material for: An Integrated mHealth App for Smoking Cessation in Black Smokers With Anxiety: Protocol for a Randomized Controlled Trial
Source: JMIR Res Protoc. 2022 May 30;11(5):e38905. doi: 10.2196/38905 (PMC9153912; doi:10.2196/38905)
Supplement: Multimedia Appendix 1 [file resprot_v11i5e38905_app1.pdf]

SUMMARY STATEMENT
(Privileged Communication)

PROGRAM CONTACT:
Derrick Tabor
301-594-8950
tabord@ncmhd.nih.gov

Release Date: 07/28/2020
Revised Date:

Application Number: 1 U54 MD015946-01

Principal Investigator

OBASI, EZEMENARI M

Applicant Organization: UNIVERSITY OF HOUSTON

Review Group: ZMD1 XLN (A1)
National Institute on Minority Health and Health Disparities Special Emphasis Panel
NIMHD Research Centers in Minority Institutions (RCMI) (U54 Clinical Trials Optional)

Meeting Date: 07/06/2020
Council: AUG 2020
Requested Start: 09/01/2020
RFA/PA: MD20-006
PCC: 20006DT

Project Title: HEALTH Center for Addictions Research and Cancer Prevention

SRG Action: Impact Score:20
Next Steps: Visit https://grants.nih.gov/grants/next_steps.htm
Human Subjects: 30-Human subjects involved - Certified, no SRG concerns
Animal Subjects: 10-No live vertebrate animals involved for competing appl.
Gender: 1A-Both genders, scientifically acceptable
Minority: 2A-Only minorities, scientifically acceptable
Age: 3A-No children included, scientifically acceptable

Project Year	Direct Costs Requested	Estimated Total Cost
1	1,823,424	2,803,230
2	1,499,997	2,306,012
3	1,499,997	2,306,012
4	1,499,997	2,306,012
5	1,499,997	2,306,012
TOTAL	7,823,412	12,027,277

1U54MD015946-01 Obasi, Ezemenari

RESUME AND SUMMARY OF DISCUSSION: This new multicomponent center grant application from University of Houston (UH) was submitted in response to RFA-MD-20-006, Research Centers in Minority Institutions (RCMI) (U54 Clinical Trials Optional). The goal of the proposed Helping Everyone Achieve a LifeTime of Health (HEALTH) Center for Addictions Research and Cancer Prevention is to advance institutional infrastructure for pursuit of cutting-edge health-equity science.
Research Project 1 aims to refine an Integrated Mobile Anxiety Sensitivity Program for Smoking (MASP) application to improve access to cigarette cessation treatment among African American (AA) Smokers. The study is very significant as AA smokers have elevated smoking rates compared to their white counterparts and are more likely to use menthol cigarettes, which are associated with a host of flavor-specific negative health outcomes. The investigative team at both UH and University of Oklahoma Health Sciences Center (OUHSC) is exceptionally qualified with complementary expertise in tobacco research and technology-based intervention, and stress-related psychopathology. The Multiple PI plan is well-articulated and reinforces the investigative team to advance the specific aims of the proposed research project. Another major strength is using a culturally adapted MASP App that is well-delineated and framed within the cultural context of interoceptive stress among AA smokers that is supported by theory and solid preliminary data. Other strengths include sound study design, measurement strategy, assessment, randomization, recruitment, dissemination, and tailored intervention in real-time based on current symptoms and experiences for AA smokers. The UH research environment is strong with a number of centers and institutes, and the consortium relationship with the OUHSC is well-suited to enhance the research capacities to support the proposed project. If successful, outcomes will make significant contribution to addiction science in understanding the social determinants of smoking and will provide policies to guide tobacco cessation intervention among AAs. However, the power analysis and conceptual innovation model are not well-defined. The focus on tobacco use is in general rather than simply smoking. Overall, the project is relevant to health disparities research and was rated in the Outstanding to Exceptional range with negligible weaknesses.
Administrative Core (AC) is well structured to provide leadership, oversight, and centralized management of all the strategic planning, research, dissemination, and implementation activities conducted by the RCMI Cores, Research Project, Pilot Grants, and administrative support staff. The AC is led by highly qualified team with strong leadership plan, expertise, and experience in managing large projects. UH is designed as the important Minority-Serving Institution to support the most ethnically diverse research and will provide the resources to address research infrastructure challenges faced by underrepresented minority scientists. Alignment of this RCMI with UH strategic priorities and the approach to creating equity are significant given UH and urban Houston context. HEALTH Research Institute (HRI) has past record in this area from which to leverage into the newly proposed RCMI Center. The AC will continue the collaborative partnerships between internal and external stakeholders to solve community-identified health challenges. The comprehensive Specific Aims are well-delineated and will be enhanced through the proposed HRI. The evaluation plan is also well-articulated with logic models, metrics and benchmarks in place. There is also a suitable plan designed to provide sustainable approach for preventing COVID-19 and chronic disease disparities. Another strength is the use of Standard Operating Procedures to guide the RCMI operations, including the centralization of fiscal and executive functions. However, the community engagement is not clearly integrated with the AC, but this element will be critical to success. The evaluation logic model is not readable. The Research Infrastructure Core (RIC) is proposed to leverage the research infrastructure to provide tremendous opportunities to engage more scientists in innovative health-equity science. The institutional commitment to support research infrastructure is strong. The RIC leadership is strong and has extensive experiences to accomplish goals for the Core and the various core facilities. There are strong RIC facilities, including Health Research Institute and the existence of a community partnership for collaboration on community-based research activities, to support investigators and research projects in the focus area. The RIC is well-integrated with other cores. Another major strength is a well-articulated Health Research Model. The elegant framework will help to advance research in a synergistic way. The

plan to deliver state-of-the-art core services in the five areas are laid out well. The evaluation metrics and timetable are well-defined. However, the sustainability of core facilities is not well-articulated. The Investigator Development Core (IDC) will enhance the scientific impact of this newly proposed RCMI Center at the UH by providing opportunities, support, and mentoring for the next generation of researchers to pursue disparities research in addictions and cancer prevention. Many strengths include capable leadership team, innovative Pilot Grant Program (PGP), strong mentorship program, clear evaluation plan with timeline and metrics, and efficient resource incubator for senior postdoctoral fellows and ESIs underrepresented in the health sciences to carry out innovative pilot research projects. Selecting the process for pilot projects is clearly described. Outcome targets are emphasized on the success of grants as well as scientific productivities. Another strength is that IDC will foster integrative support involving other cores and Research Projects to ensure the efforts are synergistic and result in a clear return on investment. However, there was insufficient information on how mentors will be recruited and incentivized. The use of internal members for the Scientific Review Group (SRG) as opposed to some external is not clearly delineated. The Community Engagement Core (CEC) will leverage and expand existing infrastructure and ongoing community partnerships to serve as the hub for community-engaged research. CEC leadership team is highly qualified and has requisite expertise to conduct a community need assessment and recruit a bilingual certified Community Health Worker to increase community access and lead community engagement research. The academic-community partnerships model is well-articulated and includes both community advisory board and community research advisory board to facilitate sustainable relationship between resources and community, stakeholders, and community members. Dissemination and translational plans are also well-designed for rapid dissemination and implementation of research findings, and for enhancing the translation of research into practice. The established facilities for community-based participatory research are adequate. The timelines and evaluation metrics are clearly defined. However, the operationalization of the proposed activities is not clearly delineated. Inclusion of grass roots community members and patients is not described in detail. There was concern for how the CEC will overcome obstacles to the community engagement. Overall, all the four cores are well-articulated to provide the needs of the Center. Some negligible weaknesses were noted for CEC innovation and for integration of the AC and CEC. All the cores as a whole are rated outstanding.

Overall, this center application is to establish the RCMI at the University of Houston to reduce health disparities in highly diverse Houston. The proposed Center includes an outstanding Research Project, four outstanding Cores, a great team, good institutional infrastructure, excellent community partnerships, a solid research training program, and great mHealth approaches in pursuing cutting-edge health-equity science. Institutional resources are exceptional and there are synergies between the project, cores and institution. This outstanding center application is assessed as high impact.

COMPONENT IMPACT SCORES
All Cores: 21
Research Project 1: 19

DESCRIPTION (provided by applicant): The proposed RCMI HEALTH Center for Addictions Research and Cancer Prevention is designed to establish a national exemplar for how community-engaged research can accelerate scientific breakthroughs that can be rapidly disseminated and implemented directly into the targeted community by trained laypersons or paraprofessionals. This will be achieved by the successful completion of five specific aims: Aim 1. Execute a centralized Research Infrastructure Core that will enhance scientific rigor, productivity, and impact of health-equity science through five primary services: (1) Research Methodology; (2) Laboratory Techniques and Facilities; (3) Data Management and Biostatistics; (4) Health Informatics; and (5) Responsible Conduct of Research, Ethics, and Compliance in health-disparities research; Aim 2. Execute a group mentoring program in the Administrative Core that provides data-driven career enhancement activities for underrepresented minority (URM) postdoctoral fellows and assistant professors pursuing careers in health-equity science; Aim 3. Strategically increase the application and success of investigators – underrepresented in the

health sciences – securing competitive NIH research grants through the Pilot Grant Program and Innovation Research Talks administered by the Investigator Development Core; Aim 4. Leverage the Community Engagement Core to facilitate equitable, collaborative, and sustainable partnerships with community members, organizations, and stakeholders to enable a bidirectional “exchange of information” that advances the potential impact of research findings for achieving health equity; and Aim 5. Promote research on minority health and health disparities by disseminating RCMI outcomes through publicly available peer-reviewed publications, presentations, white papers, policy briefs, and other materials, activities, or services disseminated into the community. This transformative infrastructure – in partnership with UH administrators, community members, stakeholders, organizations, and elected officials – provides a sustainable data-driven approach for saving lives and preventing addictions and cancer from disproportionally afflicting marginalized and underserved communities in metropolitan Houston and beyond.

PUBLIC HEALTH RELEVANCE
The diversity, population size, and health challenges of metropolitan Houston provides an ideal setting for a NIMHD Research Centers in Minority Institutions (RCMI). The University of Houston (UH) proposes to establish the Helping Everyone Achieve a LifeTime of Health (HEALTH) Center for Addictions Research and Cancer Prevention to advance institutional infrastructure to pursue cutting-edge health-equity science. Investing in health-equity science has the potential to save lives and improve the overall quality and length of life for some of our most vulnerable populations.

CRITIQUES: The written critiques of individual reviewers are provided in essentially unedited form below. These critiques were prepared prior to the meeting and may not have been revised afterwards. The “Resume and Summary of Discussion” above summarizes the final opinions of the committee.

CRITIQUE 1

OVERALL
Significance: 1
Investigator(s): 1
Innovation: 2
Approach: 2
Environment: 1

Overall Impact

This application is to establish the RCMI at the University of Houston (UH). The environment for this proposed Center at UH is very strong, and the need for research and programs to reduce health disparities in highly diverse Houston is great. The overall application summary is well written and clear. The cores are overall very strong, and generally well-integrated. The investigative team is diverse, has strengths in design, methods, statistics, community engagement, and experience in related studies and the local context. Some weaknesses in the CEC core, mainly around lack of novel approaches to building connections and communicating with the community were noted. Overall, strengths greatly outweigh weaknesses in this overall, potentially highly impactful, Center application.

1. Significance

Strengths

- Strong case made for the suitability of RCMI at UH and in Houston’s diverse urban health context.

Weaknesses

- None noted.

2. Investigator(s)

Strengths

- PI, Dr. Obasi, is well-suited to the leadership role.
- Strong biostatistics co-Is.
- Strong core and research project leads.

Weaknesses

- None noted.

3. Innovation

Strengths

- Incubator component is innovative.
- Pursuit of big health equity questions is innovative.

Weaknesses

- CEC, while a strength, does not use novel methods to increase community engagement.

4. Approach

Strengths

- SMART goals are a strength.
- Integration of RCMI into existing infrastructure increases odds of overall success.
- Good discussion of other considerations, barriers and solutions.

Weaknesses

- Some aspects of the specific cores and how they will work together not described in detail.
- Not clear how cores will share/manage knowledge as project develops.

5. Environment

Strengths

- Outstanding environment overall at UH in diverse urban Houston setting.

Weaknesses

- None noted.

Study Timeline

Strengths

- Appropriate.

Weaknesses

- None noted.

Protections for Human Subjects

Acceptable Risks and/or Adequate Protections

- Acceptable

Data and Safety Monitoring Plan (Applicable for Clinical Trials Only):

Acceptable

Inclusion Plans

- Sex/Gender: Distribution justified scientifically
- Race/Ethnicity: Distribution justified scientifically
- For NIH-Defined Phase III trials, Plans for valid design and analysis:
- Inclusion/Exclusion Based on Age: Distribution justified scientifically
- Appropriate

Vertebrate Animals

Not Applicable (No Vertebrate Animals)

Biohazards

Not Applicable (No Biohazards)

Select Agents

Not Applicable (No Select Agents)

Resource Sharing Plans

Acceptable

Authentication of Key Biological and/or Chemical Resources

Not Applicable (No Relevant Resources)

Budget and Period of Support

Recommend as Requested

ALL CORES

Overall Impact

The Cores include 1) Administrative, 2) Research Infrastructure, 3) Investigator Development, and 4) Community Engagement. Overall, the presentation of cores, investigative teams, plans and approaches, significance were all very good. Some weaknesses were noted in innovation for the CEC and for integration of the AC and CEC. However, strengths greatly outweigh weaknesses for the Cores overall.

1. Administrative Core

Strengths

- Highly significant to create the RCMI given UH and urban Houston context.
- Approach to creating equity is significant in local context.
- Alignment of RCMI with UH strategic priorities is a strength.
- Leadership plan is strong.

Weaknesses

- Community engagement strategies are not especially innovative and not clearly integrated with the AC, but this element will be critical to success.

2. Research Infrastructure Core

Strengths

- Health Research Model is a strength.
- Strong team overall.
- Well integrated with other cores.

Weaknesses

- No substantial weaknesses noted.

3. Investigator Development Core

Strengths

- Strong overall core to support mission of RCMI.
- Pilot Grant Program (PGP) is a major strength.
- Core likely to support early stage investigators in Center.
- Likely to increase scientific productivity of affiliated investigators.
- One of strongest overall components of the application.

Weaknesses

- None noted.

4. Community Engagement Core

Strengths

- CEC Director and co-Is are very strong.
- Inclusion of CAB is a strength.
- Academic-community partnership model is a strength.
- Approach is well described and there are multiple strategies to increase engagement.
- Dissemination and translational elements are strong.

Weaknesses

- Details on the theory underlying CEC is not described in detail.
- Not clear how the CEC will overcome obstacles to engagement, such as wealth disparities, transportation, lack of time, etc.
- Some strategies used in other innovative community engagement, such as creating a distinctive and attractive program identity, communicating shared values with community, not described in detail.
- Not clear what the communication plan is to increase awareness of CEC and the Center as a whole.

RESEARCH PROJECT 1
Significance: 1
Investigator(s): 1
Innovation: 2
Approach: 2
Environment: 1

Overall Impact

This project uses a mobile tool, MASP, to reduce disparities in smoking cessation among African Americans. Attention to stress/anxiety as factors in cessation with the priority population is a strength, but the innovation section lacks details on theory/conceptual model. The project has a strong investigative team with successful preliminary studies. The design, measurement strategy, recruitment, randomization/assessment, and protocol overall are sound. Description of MASP is very detailed and strong. One minor weakness was a lack of detailed power analysis. Overall, strengths outweigh minor weaknesses and the project has potential for high impact.

1. Significance

Strengths

- Disparities in African American smoking cessation compared to other groups is significant.
- Use of mHealth solution in this context is significant.

Weaknesses

- None noted.

2. Investigator(s)

Strengths

- Excellent team overall.
- Well integrated into the RCMI and other cores.

Weaknesses

- None noted.

3. Innovation

Strengths

- MASP is highly innovative.

- Approaches described to address smoking cessation disparities are innovative.

Weaknesses

- No detailed discussion of relevant theory.
- No conceptual model provided.

4. Approach

Strengths

- The design, measurement strategy, recruitment, randomization/assessment, and protocol overall are sound.
- Data analysis strategy is detailed and strong.
- Description of MASP is very detailed and strong.

Weaknesses

- No detailed power analysis provided.

5. Environment

Strengths

- Excellent overall.

Weaknesses

- None noted.

Study Timeline

Strengths

- Appropriate.

Weaknesses

- None noted.

Protections for Human Subjects

Acceptable Risks and/or Adequate Protections

- Acceptable

Data and Safety Monitoring Plan (Applicable for Clinical Trials Only):

Acceptable

Inclusion Plans

- Sex/Gender: Distribution justified scientifically
- Race/Ethnicity: Distribution justified scientifically
- For NIH-Defined Phase III trials, Plans for valid design and analysis: Scientifically acceptable
- Inclusion/Exclusion Based on Age: Distribution justified scientifically
- Acceptable

Vertebrate Animals

Not Applicable (No Vertebrate Animals)

Biohazards

Not Applicable (No Biohazards)

Select Agents

Not Applicable (No Select Agents)

Resource Sharing Plans

Acceptable

Authentication of Key Biological and/or Chemical Resources

Not Applicable (No Relevant Resources)

CRITIQUE 2

OVERALL IMPACT
Significance: 2
Investigator(s): 2
Innovation: 2
Approach: 2
Environment: 2

Overall Impact

The proposed center will focus on addictions research and cancer prevention to address public health crises caused by health disparities. Long standing community partnerships and city initiatives will be leveraged to achieve the goals of this proposal. The proposed center will create an infrastructure to promote scientific rigor and productivity, implement a mentoring program for ESI, strategically increase the success of investigators, promote sustainable partnerships and research on minority health.

1. Significance

Strengths

- Responsive to community needs for strategies to increase identification and treatment of addictions and cancer prevention in minority populations in the local community.
- Builds on existing community partnerships.

Weaknesses

- Lack of clear delineation between existing center and the one proposed in the proposal.

2. Investigator(s)

Strengths

- Strong PI with the experience to lead the team.
- Strong group of investigators with complementary skill sets.

Weaknesses

- None noted.

3. Innovation

Strengths

- Use of NIMHD's research framework to guide center.
- Multiple community boards.
- Intellectual incubator will help with success.

Weaknesses

- None noted.

4. Approach

Strengths

- Strong evaluative component with metrics and benchmarks.
- Centralized management of functions will make for efficient processes.
- Inclusion of community health workers and community liaisons will help ensure community needs and voice are present and met.
- Overall organizational structure has clear delineation of duties for each core.
- Well defined operating procedures.

Weaknesses

- None noted.

5. Environment

Strengths

- Strong track record of community engagement will help the research process.
- Alignment with city government initiatives.

Weaknesses

- Not clear how proposed renovations enhance this application. Seems more applicable to future research.

Study Timeline

Strengths

- Seems doable.

Weaknesses

- None noted.

Protections for Human Subjects

Acceptable Risks and/or Adequate Protections

- Plans adequately describe potential risks, protections, benefits, and importance of knowledge derived.

Data and Safety Monitoring Plan (Applicable for Clinical Trials Only):

Not Applicable (No Clinical Trials)

Inclusion Plans

- Sex/Gender: Distribution justified scientifically
- Race/Ethnicity: Distribution justified scientifically
- For NIH-Defined Phase III trials, Plans for valid design and analysis: Scientifically acceptable
- Inclusion/Exclusion Based on Age: Distribution justified scientifically
- Plans target African Americans as is appropriate.

Vertebrate Animals

Not Applicable (No Vertebrate Animals)

Biohazards

Not Applicable (No Biohazards)

Select Agents

Not Applicable (No Select Agents)

Resource Sharing Plans

Acceptable

- Plans are in place to share data.

Authentication of Key Biological and/or Chemical Resources

Not Applicable (No Relevant Resources)

Budget and Period of Support

Recommend as Requested

- Budget seems reasonable to complete this project.

ALL CORES

Overall Impact

Overall, the cores are well aligned to provide the needs of the project. The Administrative Core will provide strong leadership, centralized management, compliance monitoring, and oversight. The Community Engagement Core builds upon the broad-based coalition of community. The Investigator Development Core manages the pilot grant incubator program and provides enrichment activities for ESI. Finally, the Research Infrastructure Core has designed activities that support an enhanced research infrastructure.

1. Administrative Core

Strengths

- Centralization of fiscal and executive functions will create efficiency.
- Clear evaluation plan with logic models, metrics and benchmarks.
- Leadership expertise and experience with managing large projects.

Weaknesses

- None noted.

2. Research Infrastructure Core

Strengths

- Advancing the research infrastructure to promote increased research.
- Framework helps to advance work in synergistic way.
- Core leadership has skill set to accomplish goals.
- Clear evaluation metrics and timetable.

Weaknesses

- None noted.

3. Investigator Development Core

Strengths

- Innovative grant development incubator program.
- Grant proposals accepted from across campus.
- Strong mentorship program.
- Clear timeline and evaluation metrics.

Weaknesses

- None noted.

4. Community Engagement Core

Strengths

- Community advisory board and community research advisory board increases community voice.
- Use of community health workers and community liaisons increases community access and voice.
- Clear timelines and evaluation metrics.

Weaknesses

- Lack of inclusion of grass roots community members/patients.

RESEARCH PROJECT 1
Significance: 1
Investigator(s): 2
Innovation: 1
Approach: 2
Environment: 1

Overall Impact

Tobacco use is significantly higher in African American individuals and specifically the use of menthol cigarettes, which are associated with negative health outcomes. This proposal aims to refine and comprehensively culturally adapt an initially tested novel, mobile intervention that targets AS (anxiety sensitivity) among African American smokers (Mobile Anxiety Sensitivity Program for Smoking: MASP).

1. Significance

Strengths

- Tobacco use is a significant health problem facing African American smokers.
- MASP is an EBI that can be tailored for African American patients.

Weaknesses

- None noted.

2. Investigator(s)

Strengths

- Builds on prior research.
- Strong team.

Weaknesses

- Limited involvement of ESI in this project.

3. Innovation

Strengths

- Culturally adapted smoking cessation app has real value in the local community and beyond.
- Use of a transdiagnostic treatment target advances personalized medicine.

Weaknesses

- None noted.

4. Approach

Strengths

- Methods clearly described.

- Community engagement critical to project.
- Timeline clearly defined.

Weaknesses

- Data heavy study leads to questions of subject burden.

5. Environment

Strengths

- Community engagement fosters study recruitment.
- Adequate facilities to conduct the research.

Weaknesses

- None noted.

Study Timeline

Strengths

- Clear and doable.

Weaknesses

- None noted.

Protections for Human Subjects

Acceptable Risks and/or Adequate Protections

- Protections adequately described.

Data and Safety Monitoring Plan (Applicable for Clinical Trials Only):

Acceptable

- o Plans are well thought out and doable.

Inclusion Plans

- Sex/Gender: Distribution justified scientifically
- Race/Ethnicity: Distribution justified scientifically
- For NIH-Defined Phase III trials, Plans for valid design and analysis: Not applicable
- Inclusion/Exclusion Based on Age: Distribution justified scientifically
- Inclusion of only African Americans justified.

Vertebrate Animals

Not Applicable (No Vertebrate Animals)

Biohazards

Not Applicable (No Biohazards)

Select Agents

Not Applicable (No Select Agents)

Resource Sharing Plans

Acceptable

- Will disseminate findings developed by this research.

Authentication of Key Biological and/or Chemical Resources

Not Applicable (No Relevant Resources)

Budget and Period of Support

Recommend as Requested

CRITIQUE 3
OVERALL IMPACT
Significance: 3
Investigator(s): 2
Innovation: 3
Approach: 3
Environment: 1

Overall Impact

The University of Houston (UH) proposes to establish the Helping Everyone Achieve a LifeTime of Health (HEALTH) Center for Addictions Research and Cancer Prevention to advance institutional infrastructure to pursue cutting-edge health-equity science. Investing in health-equity science can save lives and improve the overall quality and length of life for some of our most vulnerable populations. The purpose of the proposed RCMI HEALTH Center for Addictions Research and Cancer Prevention is to establish a national exemplar on how community-engaged research can accelerate scientific breakthroughs. The scientific breakthroughs can be rapidly disseminated and implemented directly into the targeted community trained laypersons or paraprofessionals. The score driving factors were public health significance and rating on scientific premise for and scientific rigor of the Center and its organization, the investigative team, innovation, research environment, the strengths of the Cores, and the scientific merits of the proposed research project.

1. Significance

Strengths

- There are institutional support and investments to make a measurable and sustainable impact on local Black and Latinx communities.
- The RCMI SMART Goals summarized in Table 1 of the application.
- The health needs of African Americans and Latinx in the Houston area require attention. The proposed RCMI Center intends to address the health disparities in the catchment area and beyond.

- Leveraging existing infrastructure, in a coordinated way, to mitigate health disparities in a highly racial diverse community – City of Houston.
- Relative to the rest of the nation, Latinx and Black Houstonians have higher rates of cervical cancers (14.2 and 12.0 vs. 7.6). Likewise, Black Houstonians have higher prostate, colorectal, lung, and bronchial cancers rates relative to the rest of the nation, and higher rates of death from these cancers. Recent reports indicate “cancer clusters” with higher than expected rates of esophagus, larynx, lung, and bronchus cancers in lower-income Black neighborhoods in Houston.

Weaknesses

- Figure 1 is not readable – hence could not understand the HEALTH Research Model.

2. Investigator(s)

Strengths

- Excellent investigators with strong scholarship track record.
- Several of the investigators have collaborated on funded projects in the past.

Weaknesses

- The composition of the team is weighted heavily in the psychology and related space. To take a broad multidisciplinary approach, the team needs diversity in professional disciplines.
- All RCMI faculty (~80% from communities underrepresented in the health sciences) have substantial experience with large federal, state, and foundation research and dissemination grants in addictions and cancer prevention.

3. Innovation

Strengths

- Intellectual incubator developing new talent is innovative.
- The proposed CAB and CRAB. The CAB will participate in strategic planning, driving RCMI action plans. The CRAB will advise RCMI researchers throughout all aspects of the research process (e.g., proposal, design, execution, implementation, dissemination) with an emphasis on maximizing inclusion, reach, and impact.

Weaknesses

- The proposed methods are necessary for the success of the proposed center, but not quite innovative.

4. Approach

Strengths

- Stakeholders' input in terms of having a single research focus for the proposed RCMI Center led to the conclusion that the focus should be behavioral sciences, which would fit more URM scientists at UH.

- RCMI Leadership Team, CAB, and relevant community stakeholders will engage in intentional, strategic planning activities (120 min, every two months) that actively drive synergistic interactions across all RCMI components and research.
- Direct and Regular Communication with Dr. Amr Elnashai (VP for Research & Technology Transfer).
- Active Engagement with Eloise Brice (VP for University Advancement) to Financially Sustain the RCMI and Advance Stakeholder Relationships.
- The proposed RCMI will leverage a rare broad-based coalition that can serve as a national exemplar and training facility for developing culturally-informed and sustainable solutions to community-identified health challenges.

Weaknesses

- None noted.

5. Environment

Strengths

- Excellent Research environment.
- Strong institutional commitment - The established Health Research Institute is an interdisciplinary, University-wide research institute within the Chancellor's Healthy Community Initiative. Letters of commitment from Chancellor and President, Vice Chancellor/Vice President for Research and Technology Transfer, and Vice-Chancellor/Vice President for University Advancement.
- Strong Addiction and behavioral Research infrastructure.
- Provide excellent research resources for both community partners and researchers.
- Strong community and City and State leaders- Letters of Support from State Rep – District 147, and other community and organizational leaders.

Weaknesses

- None noted.

Protections for Human Subjects

Acceptable Risks and/or Adequate Protections

Data and Safety Monitoring Plan (Applicable for Clinical Trials Only):

Not Applicable (No Clinical Trials)

Inclusion Plans

- Sex/Gender: Distribution justified scientifically
- Race/Ethnicity: Distribution justified scientifically
- For NIH-Defined Phase III trials, Plans for valid design and analysis: Not applicable
- Inclusion/Exclusion Based on Age: Distribution justified scientifically

Vertebrate Animals

Not Applicable (No Vertebrate Animals)

Biohazards

Not Applicable (No Biohazards)

Select Agents

Not Applicable (No Select Agents)

Resource Sharing Plans

Acceptable

Authentication of Key Biological and/or Chemical Resources

Not Applicable (No Relevant Resources)

Budget and Period of Support

Recommend as Requested

ALL CORES

Overall Impact

Overall, for each of the proposed cores, the strengths outweigh the weaknesses. Seasoned researchers lead the administrative with administrative experience. Additionally, the core is well-positioned to facilitate and manage the interactions and linkages between the cores. The Core Facilities are excellent with excellent leadership, and the proposed retrofit of the lab space will support the research focus of the proposed center and research project. The IDC proposes to work collaboratively with the Administrative Core (AC), Community Engagement Core (CEC), Research Infrastructure Core (RIC), and the Research Project PI to ensure the efforts are synergistic and result in a clear return on investment. The proposed research activities and mentoring plan are quite ambitious. The proposed center has an excellent community engagement core dedicated to fostering a bi-directional relationship with the community and actively involving the community in the proposed research. Overall, the research cores are outstanding and structured to support Addictions Research and Cancer Prevention.

1. Administrative Core

Strengths

- Strong and experienced leadership and researchers.
- UH is committed to addressing the needs of a highly diverse population - the most ethnically diverse research institution, a designated Hispanic Serving Institution, and Asian American and Native American Pacific Islander-Serving Institution.

- The NIMHD RCMI at UH will provide the quintessential resources needed to address University research infrastructure challenges faced by URM scientists, while also accelerating the execution of bold health-equity research across 12 Colleges and 28 academic units.
- The past track record of HEALTH Research Institute (HRI) is being leveraged into the newly proposed RCMI Center. The HRI leveraged ~$600K in administrative support and M&O to secure six extramural grants: (1) NCI P20 [$1.27M]; (2) NCI R03 [$100K]; (3) NIDA R21 [$275K]; (4) CPRIT [$1.35M]; (5) CPRIT [$291K], and (6) United Health Foundation [$2M] – totaling over $5.26M and demonstrating an accelerated and bold return on investment in addiction and cancer prevention research.
- The RCMI will foster collaborative partnerships between internal and external stakeholders to solve community-identified health challenges.
- Use of Standard Operating Procedures to guide the RCMI operations, including organizational structure, purchasing, accounting/budgetary controls, payroll distribution, travel, consulting, property management, ethics, and conflicts of interest.
- Program Evaluation Plan adequately described.
- Description of the coordination and interaction between the CEC, IDC, and RIC.
- Persuasive letters of support from advisory committee members.

Weaknesses

- Lack of insight into the challenges faced by URM scientists at UH.
- Given the current research infrastructure at UH, and the RCMI funding mechanism is to support infrastructure building, the investigators did not articulate how the RCMI will enhance current infrastructure.
- RCMI Integrated Evaluation Model (Logic Model) is not readable.

2. Research Infrastructure Core

Strengths

- Excellent Core facilities to support research in the focus area.
- Strong institutional commitment to support research infrastructure.
- Strong leadership for the Core and the various core facilities.
- The scientific mission is to enhance, advance strategically, and accelerate the scientific rigor, productivity, and impact of health-equity science conducted at UH, focusing on disparities in addictions and cancer prevention.
- The RIC will serve as an intellectual incubator that brings together transdisciplinary research teams linking RCMI investigators (principally postdocs and faculty who consist of racial/ethnic minorities, women, individuals with disabilities, and those from disadvantaged backgrounds) with health scientists across the university.
- Inclusive marketing strategies are excellent for increasing the use of available resources.
- Easy access and the ability to track requests using online SharePoint.

Weaknesses

- Did not address sustainability of core facilities.

3. Investigator Development Core

Strengths

- The IDC will also work collaboratively with the Administrative Core (AC), Community Engagement Core (CEC), Research Infrastructure Core (RIC), and the Research Project PI to ensure the efforts are synergistic and result in a clear return on investment.
- Applicants for the PGP will be strongly encouraged to take advantage of the established scientific network in metropolitan Houston. The Texas Medical Center (e.g., Baylor College of Medicine, DeBakey VAMC, MD Anderson Cancer Center, Prairie View A&M, Texas A&M, Texas Southern University, and UT Health, to mention a few) to facilitate a team science approach that fosters interdisciplinary collaborations with experienced investigators.
- The IDC plans to address gaps in the URM in leadership roles in research and academia- city-data NIH Office of Scientific Workforce Diversity.
- The IDC will serve as a resource-efficient incubator for postdocs and assistant professors underrepresented in the health sciences to generate preliminary data to be successfully leveraged into developmentally appropriate NIH funding and professional growth in health-equity science.
- Inclusive Team Science that Advances a More Diverse Faculty and Scientific Review Process.
- Strong leadership team to guide and provide mentorship.
- UH will build on the experience of providing pilot project funding to new and early career faculty and postdocs.
- Well described process of applying and receiving pilot grant funding.
- Mentoring Program aimed at transitioning Pilot Grants into funded NIH Research Projects.

Weaknesses

- Not sure how the mentors will be recruited and the criteria for being a mentor.
- There was not adequate information or articulation of how mentors will be incentivized.
- The investigators did not address using internal members for the Scientific Review Group (SRG) as opposed to some external, mainly if the expertise is not present internally or if there is the person internally but is in conflict.

4. Community Engagement Core

Strengths

- The CEC will leverage and expand existing infrastructure and ongoing community partnerships to serve as the hub for community-engaged research at the University of Houston, the second most ethnically diverse major research university in the U.S.
- Provision of Facilities & Resources for Community Stakeholders.

- The CEC support for Community-engaged Research.
- Strong leadership for the CEC.
- Dr. Leal, a Qualitative Scientist and bi-lingual (Spanish), will conduct a community needs assessment.
- Recruitment of a bi-lingual certified Community Health Worker.
- The CEC will address the following critical aspects of addressing health disparities: (1) Community Omission from Research Creates and Maintains Health Disparities; (2) Community Omission from Research Maintains the Translation Gap; and (3) Academic-Community Partnerships are Well Placed to Achieve Health Equity.
- The CEC will serve as the Hub of Academic-Community Partnerships.
- Persuasive letters of support from the community.
- Activity Map of project CEC Service Utilization across the Funding Period.
- The community's ability to seek support services from the CEC and RCMI Center via an online request form.

Weaknesses

- Lack of details on the operationalization of the proposed activities.
- Lack of clarity of community involvement in the research continuum, from concept to dissemination.

RESEARCH PROJECT 1
Significance: 2
Investigator(s): 2
Innovation: 3
Approach: 2
Environment: 1

Overall Impact

Tobacco use is the leading cause of preventable death and disability and results in a substantial economic burden. Estimates suggest that 13.7% of adults in the United States (US) are current smokers. However, the distribution of tobacco use is uneven in society. African American smokers evince elevated smoking rates (14.6%) and are significantly more likely to use menthol cigarettes associated with a host of flavor-specific, resulting in adverse health outcomes. The present proposal aims to refine and comprehensively culturally adapt an initially tested novel mobile intervention targets AS among African American smokers (Mobile Anxiety Sensitivity Program for Smoking: MASP). The proposed intervention, framed within the cultural context of interoceptive stress among African American smokers, is supported by theory, empirical evidence, and characteristics of this group. The score driving factors were public health significance and rating on scientific premise for and scientific rigor of the Center and its organization, the investigative team, innovation, research environment, the strengths of the Cores, and the scientific merits of the proposed research project.

1. Significance

Strengths

- A major contributing factor to smoking among African Americans appears to be their increased exposure to interoceptive-stress symptoms.
- African American smokers smoke fewer cigarettes per day and tend to begin smoking later than national norms. Nevertheless, African Americans evince higher levels of nicotine dependence and serum cotinine.
- African Americans suffer disproportionately from tobacco-related disease and death and have a higher incidence and mortality rate from lung cancer compared to European Americans. This issue is alarming, as the current prevalence rate of smoking among African Americans is 14.6%,32, which reflects approximately 6,421,678 million persons.
- African Americans are a health disparity group for interoceptive problems, including somatic symptoms, anxiety, stress, and pain, and evince stronger relations between negative emotional states and somatic experiences compared to European Americans. African Americans diagnosed with anxiety disorders experience higher rates of hypertension, a condition for which African Americans are almost twice as likely to be diagnosed than European Americans.
- The investigative provide a strong scientific premise for the proposed research.
- The proposed research is the next step in the progression of the research aimed to refine and comprehensively culturally adapt an initially tested novel mobile intervention that targets AS among African American smokers (Mobile Anxiety Sensitivity Program for Smoking: MASP).

Weaknesses

- None noted.

2. Investigator(s)

Strengths

- The Project PI, Dr. Zvolensky, is a Distinguished Professor in the Department of Psychology at the University of Houston and Director of the Anxiety and Health Research Laboratory and Substance Use Treatment Clinic. The PI has published over 700 peer-reviewed articles, books, and book chapters. The publications cover the co-occurrence of anxiety and stress-related psychopathology.
- Co-investigator, Dr. Garey, is a Research Assistant Professor in the Department of Psychology at the University of Houston. Her research employs advanced methodological designs to elucidate the risk and protective factors of addictive behavior and treatment outcomes. Dr. Garey has published over 100 peer-reviewed articles and books/book chapters on topics within her area of research and served as PI on several federally and privately funded grants, including a stage I trial of a technology-based intervention for mood and substance use.
- Dr. Gallagher, co-I and biostatistician, is an Associate Professor in the Department of Psychology and the Texas Institute for Measurement, Evaluation, and Statistics at the University of Houston. He has published over 110 peer-reviewed articles and book chapters primarily focusing on transdiagnostic treatments, mechanisms of change, and resilience factors for emotional disorders.
- The other Co-Investigators are: Dr. Vujanovic is an Associate Professor of Psychology and Director of the Trauma and Stress Studies Center at the University of Houston. She has published over 160 peer-reviewed articles and book chapters primarily focusing on trauma,

stress, and substance misuse among high-risk populations (e.g., first responders, human trafficking victims). Dr. Chen, a Research Assistant Professor at the HEALTH Research Institute, University of Houston, provides statistical support on grant applications, manuscripts, and progress reports, and offers statistical support and data management on large-scale sponsored research dissemination projects associated with the HEALTH Research Institute.

- The overall investigative team at UH and OUHSC are excellent in Tobacco research; with the OUHSC's team with more expertise in tobacco research.
- The use of the MPI mechanism was justifiable and strengthened the investigative team.

Weaknesses

- Majority of the investigative team had similar training and background. It would seem a multi-disciplinary team would have been good.

3. Innovation

Strengths

- Use of a culturally adapted smoking cessation App.
- Focus on transdiagnostic treatment targets as opposed to syndromes or symptoms.
- Provision of an automated intervention that tailors treatment content in real-time, since the MASP app can significantly impact tobacco-related health disparities for African American smokers.
- This study will be among the first to (1) dynamically tailor smoking cessation treatment content in real-time based upon current symptoms and experiences for African American smokers and (2) identify culturally-relevant moderators of intervention effects to isolate for whom the intervention works best.

Weaknesses

- None noted.

4. Approach

Strengths

- Good preliminary study data that serves as the basis for the proposed study.
- The use of a tailored intervention – Treatment on a Schedule.
- Solicit the assistance of the CEC for recruitment in addition to strategies described in the application.
- The intervention components and phases were well described.
- Data collection and measurement are extensive and detailed.
- Incorporating COVID 19 questions and assessing the impact of COVID 19 later exploratory analysis is excellent.
- They are ensuring that all phones used are smartphones.

- Data analytic plan was detailed, appropriate and adequate.
- The study was adequately powered.
- The dissemination plan was adequate.

Weaknesses

- The use of the CEC, CAB, and CRAB for only recruitment retains the old paradigm of “helicopter research.” Investigators should seek input from these groups before recruitment.

5. Environment

Strengths

- Robust research infrastructure at UH.
- The consortium relationship with the University of Oklahoma Health Sciences Center (OUHSC) is a strength.
- OUHSC has an NCI-designated Stephenson Cancer Center (SCC). The NCI Designated SCC mHealth Shared Resource is led by Dr. Michael Businelle and staffed by a program coordinator, two research technicians, and 4 Master’s level Computer Scientists and Engineers. The mHealth resource offers resources that empower researchers to build, test, and launch technology-based assessment and intervention tools.

Weaknesses

- None noted.

Study Timeline

Strengths

- The timeline was adequate.

Weaknesses

- None noted.

Protections for Human Subjects

Acceptable Risks and/or Adequate Protections

Data and Safety Monitoring Plan (Applicable for Clinical Trials Only):

Acceptable

Inclusion Plans

- Sex/Gender: Distribution justified scientifically
- Race/Ethnicity: Distribution justified scientifically
- For NIH-Defined Phase III trials, Plans for valid design and analysis: Scientifically acceptable
- Inclusion/Exclusion Based on Age: Distribution justified scientifically

Vertebrate Animals

Not Applicable (No Vertebrate Animals)

Biohazards

Not Applicable (No Biohazards)

Select Agents

Not Applicable (No Select Agents)

Resource Sharing Plans

Acceptable

Authentication of Key Biological and/or Chemical Resources

Not Applicable (No Relevant Resources)

CRITIQUE 4
OVERALL
Significance: 2
Investigator(s): 1
Innovation: 3
Approach: 1
Environment: 1

Overall Impact

The University of Houston proposes RCMI Health Center for Addiction Research and Cancer Prevention with the overall arching goal of addressing the burden of addition health disparities in a predominantly Hispanic, African American, as well as other vulnerable populations where this university is located. The proposed application is coming from an ideal research environment, University of Houston, comprising a number of centers and institutes, including Research Clinic for Substance Abuse, Culture and Health Research Center on Emotion and Adjustment, Center for Social and Policy Research, Child and Family Center for Innovative Research, to name a few. These centers and institutes are quite suited to enhance the research capacity needs to support the proposed program's research aims. Furthermore, the proposed leadership of the RCMI will be led by a well-qualified and experienced scientist possessing the requisite expertise needed to advance the specific aims of the proposed program cores and proposed research project. It is also important to note that the proposed RCMI's research model has the capacity to lend itself to NIMHD's research framework that conceptualizes health behavior and outcome as an intersection of race, gender, sexual orientation and social determinants of health matters. In view of this background, It is the overall assessment of this reviewer that the proposed center clearly has the capacity and well suited for the likelihood to exert a sustained powerful influence in reducing the burden of addiction health disparities among the target population as identified in the specific aims of the proposed Center.

1. Significance

Strengths

- Appropriate team mentoring program designed to provide data driven career enhancement activities for underrepresented doctoral fellows and junior faculty.

- Center designed to enhance research infrastructure to accelerate the university's capacity to conduct cutting-edge research in addiction science.
- The proposed center will be led by a team of two accomplished faculty (Drs. Ezemmenari Obassi and Lorari Reitzel) with exceptional research and organizational backgrounds in leading research organizations like that proposed.
- Proposed partnership with Federally qualified health center designed to integrate care facility on campus as part of the proposed center's strategy for accomplishing center's proposed aims.
- Project narrative clearly identifies the need for research infrastructure to enhance cutting edge in addiction science research aimed at reducing the burden of addition health disparities in Hispanics, African American and other vulnerable populations.
- Target population disproportionately bear the burden of addiction health disparities.

Weaknesses

- None observed.

2. Investigator(s)

Strengths

- The Center PI, as well as the PI, of the proposed research project, Dr. Ezemmenari Obasi, Ph.D., is Professor of PHLS; He is the Co-founder/director of Houston University's Health Research Institute; He is Director, Hwemudua Additions & Health Disparities Laboratory; He has received funding from CDC, CPRT, NCI & UnitedHealth Foundation. His current program of research focuses on addictions, cultural predictions of health behaviors and cancer prevention that disproportionately affects African American community. His administrative experience covers such areas as department chair, associate Dean for research and over ten years of experience managing a research lab.
- The Center's Co-PI, Dr. Lorrai Reitz, Ph.D., Professor, PHLS; Co-Founder/Co-Director, Health Research institute; director, social Determinants/Health disparities Laboratory; Has received funding from CDC, CPRIT, and NCI.

Weaknesses

- None observed.

3. Innovation

Strengths

- Proposed action plan that will be executed through synergistic interactions between all RMCI components, scientists, and community coalitions.
- Aims to execute a centralized infrastructure to enhance scientific rigor.
- Aims to facilitate equitable collaborative partnerships with community members and stakeholders designed to enhance potential impact of the center's overarching goal of reducing the burden of disparities particularly in addiction health as well as cancer burden among the target population.

Weaknesses

- None noted.

4. Approach

Strengths

- Well-designed organizational structure whereby the PI will be supported by an advisory committee as well as an advisory board.
- Well experienced RCMI leadership team consisting of RCMI PI, as well as Core Directors, including Project Core Directors, and director of the Research Core.
- Integrated approach designed to integrate all Cores as well as proposed research project designed to produce optimum output of the proposed RCM Health Center for Addiction Research and Cancer Prevention.

Weaknesses

- None observed.

5. Environment

Strengths

- The proposed application is coming from an ideal research environment, University of Houston, comprising a number of centers and institutes, including Research Clinic for Substance Abuse, Culture and Health Research Center on Emotion and Adjustment, Center for Social and Policy Research, Child and Family Center for Innovative Research, to name a few.
- Centers and institutes are quite suited to enhance the research capacity needs to support the proposed program's research aims. Furthermore, the proposed leadership of the RCMI comprises of a well-qualified and experienced scientist with requisite expertise needed to advance the specific aims of proposed program cores and proposed research project.
- Appropriate team mentoring program designed to provide data driven career enhancement activities for underrepresented doctoral fellows and junior faculty.

Weaknesses

- None observed.

Study Timeline

Strengths

- Well established timeline to implement and complement all components of the proposed study.

Weaknesses

- None observed.

Protections for Human Subjects

Acceptable Risks and/or Adequate Protections

- All elements of risk assessment have met threshold for adequate protection of human subjects.

Data and Safety Monitoring Plan (Applicable for Clinical Trials Only):

Acceptable

Inclusion Plans

- Sex/Gender: Distribution justified scientifically
- Race/Ethnicity: Distribution justified scientifically
- For NIH-Defined Phase III trials, Plans for valid design and analysis:
- Inclusion/Exclusion Based on Age: Distribution justified scientifically

Vertebrate Animals

Not Applicable (No Vertebrate Animals)

Biohazards

Acceptable

Select Agents

Not Applicable (No Select Agents)

Resource Sharing Plans

Acceptable

- Well organized resource sharing plan put in place.

Authentication of Key Biological and/or Chemical Resources

Not Applicable (No Relevant Resources)

Budget and Period of Support

Recommend as Requested

- Well defined budget and period of support.

ALL CORES

Overall Impact

All core areas of the proposed Center are well developed and are assessed to have the capacity to accomplished stated aims for each core and the Center as a whole. Among the factors that informed the overall impact score include the following:

- Well-qualified PI and Co-PIs.
- Well-organized aims designed to accomplish the overarching goal of the proposed center.

1. Administrative Core

Strengths

- The proposed center is designed to enhance the aims of the university's existing Health Research Institute.

- The administrative core provides adequate plan for strategic leadership for the RCMI.
- Adequate plan designed to provide sustainable approach for preventing infectious as well as chronic diseases disparities.

Weaknesses

- Lack of organizational structure delineating lines of authority for the administration of the center's Core areas and proposed research project.

2. Research Infrastructure Core

Strengths

- The Research Infrastructure Core of the proposed Center identifies a strong research infrastructure at university of Houston. The strengths of the identified infrastructure include the following:
  - Research institution.
  - Research meeting space for faculty and students.
  - Health Research Institute that houses a diverse research faculty.
  - A research center that addresses health disparities by leveraging a highly experienced interdisciplinary group of researchers that engage in a broad range of health disparities research activities.
  - Existence of several health research institutes.
  - Existence of a community partnership for collaboration on community-based research activities.

Weaknesses

- Lack of integration of center's budget into the overall administrative strategy of the proposed program.

3. Investigator Development Core

Strengths

- Supports grant writing mentoring activities in support of funding successes of postdoctoral fellows as well as junior faculty.
- Fosters integrative support involving other core areas, including Community Engagement, Administrative and Research infrastructure Cores.

Weaknesses

- None observed.

4. Community Engagement Core

Strengths

- Well designed for rapid dissemination and implementation of research findings.
- Established community Advisory board to facilitate sustainable relationship between resources and community, stakeholders, and community members.

- Promotes community-based solutions to health disparities in addiction designed to enhance the translation of research into practice.
- Adequately established facilities for community participatory research.

Weaknesses

- Lack of evidence for integration of community engagement core and center's overall organizational structure.

RESEARCH PROJECT 1
Significance: 1
Investigator(s): 1
Innovation: 3
Approach: 2
Environment: 1

Overall Impact

Proposed study focuses on addiction, an important health disparities research area that when successfully executed would make significant contribution to health disparities research, particularly research focusing disparities related to addiction among African Americans. The proposed research, when successful, will make significant theoretical as well as methodological contribution to addiction science in understanding the social determinants of smoking among African Americans and other vulnerable populations. Also, if successful, this study will inform policies affecting the prevention of tobacco use among African Americans.

1. Significance

Strengths

- The project's stated primary aim is to refine existing novel mobile intervention among African American smokers.
- Provides extensive literature review that points to the fact that African Americans have higher levels of smoking rates compared to their white counterparts, that African Americans are less likely to maintain cessation compared to other ethnic/ racial groups in America.
- Cessation intervention directed to African Americans might benefit from specific focus on increasing the ability to adaptively respond to interoceptive stress.
- Findings of the proposed study may improve existing lack of treatment smoking cessation among African Americans focusing on sensitivity to interoceptive stress.

Weaknesses

- Need to as well, focus on tobacco use in general rather than simply smoking.

2. Investigator(s)

Strengths

- Dr. Ezemmenari Obasi, Ph.D., is Professor of PHLS; Co-founder/director, Health Research Institute; Director, Hwemudua Additions & Health Disparities Laboratory; He is the PI of the proposed center; He has received numerous research grants, including funding from CDC, CPRT, NCI & UnitedHealth Foundation. His current program of research focuses on addictions, cultural predictions of health behaviors and cancer prevention that disproportionately affects

African American community. His administrative experience covers such areas as department chair, associate Dean for research and over ten years of experience managing a research lab.

Weaknesses

- None observed.

3. Innovation

Strengths

- Use of culturally adapted smoking cessation app.
- Intervention framed within the cultural contest of interoceptive stress among African Americans.
- Use smart phones to identify high-risk smoking lapse.

Weaknesses

- None identified.

4. Approach

Strengths

- Use of past and on-going studies to refine and evaluate the feasibility, acceptability, and efficacy of novel mobile anxiety sensitivity program for smoking apps among African American smokers.
- Integration of the proposed behavior science research project into the proposed RCMI core areas, including the program's administrative, infrastructure, community engagement cores in efforts to enhance the project's outcome.
- Well-developed qualitative and quantitative data analysis plan, including the use of latent growth multilevel, structural equation and survival models to test proposed hypothesis.
- Proposed use of multiple approaches to modeling changes in abstinence to evaluate preliminary hypothesis to evaluate the impact of administered treatment among study population.

Weaknesses

- None observed.

5. Environment

Strengths

- The proposed is located at an ideal research environment, University of Houston, comprising a number of centers and institutes, including Research Clinic for Substance Abuse, Culture and Health Research Center on Emotion and Adjustment, Center for Social and Policy Research, Child and Family Center for Innovative Research, to name a few.
- Centers and institutes are quite suited to enhance the research capacity needs to support the proposed project's research aims. Furthermore, the proposed leadership of the RCMI comprises of a well-qualified and experienced scientist with requisite expertise needed to advance the specific aims of the proposed research project.
- Proposed center's PI is exceptionally qualified

Weaknesses

- None noted.

Study Timeline

Strengths

- Well established eligibility criteria for inclusion/exclusion of women and children.
- Acceptable criteria for sources of materials.
- Acceptable criteria for potential risks.
- Acceptable activities timeline for project implementation and completion.

Weaknesses

- None observed.

Protections for Human Subjects

Acceptable Risks and/or Adequate Protections

- Acceptable criteria for potential risks

Data and Safety Monitoring Plan (Applicable for Clinical Trials Only):

Acceptable

- o Well organized and acceptable plan for data and safety monitoring.

Inclusion Plans

- Sex/Gender: Distribution justified scientifically
- Race/Ethnicity: Distribution justified scientifically
- For NIH-Defined Phase III trials, Plans for valid design and analysis:
- Inclusion/Exclusion Based on Age: Distribution justified scientifically
- Inclusion of appropriate age for study subjects has been clearly identified.

Vertebrate Animals

Not Applicable (No Vertebrate Animals)

Biohazards

Not Applicable

Resubmission

Not Applicable

Renewal

Not Applicable

Select Agents

Not Applicable (No Select Agents)

Resource Sharing Plans

Acceptable

- Acceptable resource sharing plan has been identified.

Authentication of Key Biological and/or Chemical Resources

Acceptable

Admin-Core-001 - RCMI Administrative Core
(Description as provided by applicant)
The Administrative Core (AC) will provide leadership, oversight, and centralized management of all the strategic planning, research, dissemination, and implementation activities conducted by the RCMI Cores, Research Project, Pilot Grants, and administrative support staff. The AC will provide coordination, communication, and evaluation for all RCMI components, in support of the overarching goals of (1) significantly enhancing UH's infrastructure and environment to advance health-equity science in addictions and cancer prevention research; and (2) increasing underrepresented minority (URM) scientists' productivity, discovery, and extramural funding to pursue innovative research and dissemination activities. The long-term goal of the AC is to provide an infrastructure for the RCMI to leverage health-equity science – in partnership with community members, organizations, stakeholders, and elected officials – to collaboratively implement a sustainable data-driven approach for saving lives and preventing infectious and chronic diseases from disproportionally afflicted vulnerable communities. This goal will be achieved by the successful completion of 5 Specific Aims: Aim 1: Provide strategic leadership for the RCMI – including streamlined administrative oversight, efficient operational management, clear communication, budget management/oversight, and synergistic coordination of all components – to maximize a collective impact on health-equity science. Aim 2: Institute an integrative RCMI Leadership structure that oversees synergistic planning, implementation, and progress to ensure all aims and milestones are met on time and within budget. Aim 3: Execute an evidence- based team mentoring program with data-driven career enhancement activities for postdoctoral fellows and new investigators interested in advancing health-equity science. Aim 4: Advance equitable, collaborative, synergistic, and sustainable relationships between all RCMI components, URM scientists, community members, organizations, stakeholders, and elected officials. Aim 5: Develop a centralized program evaluation system that leverages a clear logic model to inform effective resource allocations and demonstrate an accelerated RCMI return on investment. The AC will provide the support and infrastructure needed for the daily management and operations of the RCMI, maintain open lines of communication among all the associated Cores, Research Project, Pilot Projects, administrative support staff, investigators, and NIMHD personnel. This AC's management structure and evaluation plan will ensure that the aims of the RCMI are successfully achieved.

Animal Subject Code: N
Human Subject Code: N

Resrch-Infr-Core-001 - RCMI Research Infrastructure Core
(Description as provided by applicant)
The University of Houston (UH) has approximately 400 faculty (~150 from communities underrepresented in the health sciences) conducting health-related research. These faculty span 12 Colleges and 28 academic units. This recent environmental scan suggests (1) there is a natural wellspring of scientists from a broad range of health disciplines that can leverage the Research Infrastructure Core (RIC) to advance their research; and (2) there is a tremendous opportunity to engage more scientists in innovative health-equity science that can discover novel solutions to disparities in addictions and cancer prevention afflicting our underserved and marginalized communities

in Houston and beyond. The RIC will be executed by experienced scientists that are passionate about health-equity science. Ultimately, the proposed RCMI is uniquely situated to aggressively launch a centralized RIC capable of actualizing four specific aims: Aim 1. Execute a centralized research infrastructure that will enhance the scientific rigor, productivity, and impact of addictions and cancer prevention research at UH. Plan: Deliver state-of-the-art core services in the areas of (1) Research Methodology; (2) Laboratory Techniques and Facilities; (3) Data Management and Biostatistics; (4) Health Informatics; and (5) Responsible Conduct of Research, Ethics, and Compliance in health-disparities research; Aim 2. Develop an intellectual incubator that generates transdisciplinary research teams linking investigators underrepresented in the health sciences with established investigators at UH. Plan: Execute a comprehensive marketing plan that targets and encourages University leadership, faculty, and postdoctoral fellows to leverage RIC services to advance their science while taking the opportunity to build new transdisciplinary research teams that pursue novel solutions to complex health challenges; Aim 3. Provide dynamic core services for RCMI scientists that are cost-effective, accessible, efficient, and with excellent customer service. Plan: Execute a user-friendly electronic ticketing and tracking system that will monitor service utilization and satisfaction in real-time, while also informing the data-driven redistribution of resources to maximize end-user needs and demonstrate a substantial return-on-investment; Aim 4. Pursue collaborations that minimize University duplication of resources and build bridges with local and national exemplars. Plan: Work with the Directors of the local and national exemplars to engage in a shared vision that advances health-equity science. Ultimately, large-scale innovative research that has strong scientific rigor, transparency, and reproducibility cannot be fully actualized without an efficient state-of-the-art research infrastructure.

Animal Subject Code: N
Human Subject Code: N

Investig-Dev-Core-001 - RCMI Investigator Development Core
(Description as provided by applicant)
The success of the scientific community’s ongoing efforts to enhance health outcomes for health disparities populations depends greatly on our investment in inspiring and developing a new generation of scientists who work creatively on these important, complex public health problems over the coming years. Accordingly, we propose to establish an Investigator Development Core (IDC) that will enhance the scientific impact of this newly proposed Research Centers in Minority Institutions (RCMI) at the University of Houston (UH) by identifying and providing opportunities, support, and mentoring for the next generation of researchers to pursue bold and groundbreaking research addressing disparities in addictions and cancer prevention. The IDC’s primary feature is a Pilot Grant Program that will fund 3-5 seed awards annually to support innovative projects addressing disparities in addictions and cancer prevention. We will actively solicit applications on these critical scientific topics from the UH academic community, encouraging and mentoring investigators from underrepresented backgrounds to participate in this program. The IDC has a rigorous shaping, evaluation, review, and funding process that will ensure the pilot grants comply with Federal requirements and help postdoctoral fellows and assistant professors leverage these resources to support their emerging programs of research in health-equity science. To that end, pilot applicants and investigators will also receive hands-on mentoring to generate a new pipeline of health disparities researchers from communities underrepresented in the health sciences. The long-term goal of the IDC is to leverage innovative pilot grants and hands-on mentorship that support opportunities for postdoctoral fellows and assistant professors to secure career awards or developmentally appropriate research grants that facilitate their pathway towards independence. The IDC will facilitate and enhance the innovation of the RCMI by serving as a unique research resource in the region that will enhance local and national workforce development in minority health and health disparities.

Animal Subject Code: N
Human Subject Code: N

Comm-Engag-Core-001 - RCMI Community Engagement Core
(Description as provided by applicant)
Governed by the values of integrity, partnership, and equity, the Community Engagement Core (CEC) uses a broad base of community expertise and guidance that will interact with the other Cores and research projects that comprise the RCMI to enable: (1) the prioritization and conduct of research that addresses community- guided health concerns; and (2) the rapid dissemination and implementation of research findings to achieve health equity. The CEC will work closely with a Community Advisory Board (CAB) and Community Research Advisory Board (CRAB) to: (1) assess community health priorities; (2) guide research emphases and design; (3) enable and sustain a trusted and contributory presence in communities; (4) empower community members to be agents of change; and (5) guide the application of research findings toward meaningful and health- promoting community initiatives. CEC Specific Aims are to: (1) Facilitate equitable, collaborative, and sustainable relationships between researchers and community members/stakeholders to enable a bi- directional “exchange of information” that augments the potential impact of research findings for achieving health equity; (2) Build and nurture trust within communities to enable the recruitment and retention of underrepresented groups in research; and (3) Enhance the translation of research into practice through the timely and tailored dissemination of research findings to key stakeholders including community partners, health care organizations, policymakers, and the scientific community. CEC Specific Aims will be guided in inquiry and action by key recommendations to achieving community-based solutions to health challenges1 and via an Interactive Systems Framework2,3 for dissemination and implementation. The CEC will leverage and expand extant infrastructure and ongoing community partnerships to serve as the hub for community-engaged research at the University of Houston, the second most ethnically diverse major research university in the U.S.

Animal Subject Code: N
Human Subject Code: N

Project-001 - Improving Access to Cigarette Cessation Treatment Among African American Smokers: Development and Evaluation of an Integrated mHealth Application
(Description as provided by applicant)
Tobacco use is the leading cause of preventable death and disability and results in substantial economic burden. Estimates suggest that 13.7% of adults in the United States (US) are current smokers. Yet, tobacco use is not equally distributed in society. African American smokers evince elevated smoking rates (14.6%) and are significantly more likely to use menthol cigarettes, which are associated with a host of flavor-specific negative health outcomes. Additionally, African American smokers are less likely to maintain cessation compared to European American and Latinx smokers despite making more quit attempts. A major contributing factor to smoking among African Americans appears to be their increased exposure to interoceptive-stress symptoms. Consequently, cessation interventions directed toward African American smokers might benefit from specific focus on increasing the ability to adaptively respond to interoceptive stress (e.g., anxiety, bodily sensations, stress-related burden due to racism or discrimination during a quit attempt and thereafter). Notably, motives for smoking to cope with negative mood states are central to cigarette use among those with greater sensitivity to stress (i.e., higher anxiety sensitivity [AS]; a well-established malleable, transdiagnostic construct for emotional symptoms and disorders and the maintenance of smoking). Past work has not leveraged the potential of AS to better understand smoking and interoceptive stress relations among this established health disparities group. The present proposal aims to refine and comprehensively culturally adapt an initially tested novel, mobile intervention that targets AS among African American smokers (Mobile Anxiety Sensitivity Program for Smoking: MASP). Our intervention is framed within the cultural context of interoceptive stress among African American smokers, which is supported by theory, empirical evidence, and characteristics of this group. In Phase I, we will further culturally tailor our preliminarily test treatment (MASP 1.0) with African American-smoking content and history to develop

MASP 2.0, which will be pilot tested during this phase. In Phase I, 25 African American smokers with elevated AS will be administered MASP 2.0 for 6 weeks. Following completion of Phase I, we will further refine MASP 2.0 based on qualitative and quantitative data from participants and the research team to produce MASP 3.0. In Phase II, 200 African American smokers with elevated AS will be enrolled and randomly assigned to either: (1) the smartphone-based National Cancer Institute (NCI) QuitGuide app for standard mobile smoking cessation treatment (2) MASP 3.0. Participants in Phase I and Phase II will complete an in-person baseline assessment, pre- and post-quit ecological momentary assessments, an end-of-treatment qualitative interview, and follow-up assessments at weeks 1, 2 (quit date), 3, 4, 5, 6, 28, and 54 (52 weeks post-quit) via the app as well as receive nicotine replacement therapy.

Animal Subject Code: N
Human Subject Code: Y

THE FOLLOWING SECTIONS WERE PREPARED BY THE SCIENTIFIC REVIEW OFFICER TO SUMMARIZE THE OUTCOME OF DISCUSSIONS OF THE REVIEW COMMITTEE, OR REVIEWERS' WRITTEN CRITIQUES, ON THE FOLLOWING ISSUES:

PROTECTION OF HUMAN SUBJECTS: ACCEPTABLE

INCLUSION OF WOMEN PLAN: ACCEPTABLE

INCLUSION OF MINORITIES PLAN: ACCEPTABLE

INCLUSION ACROSS THE LIFESPAN: ACCEPTABLE

COMMITTEE BUDGET RECOMMENDATIONS: The budget was recommended as requested.

Footnotes for 1 U54 MD015946-01; PI Name: Obasi, Ezemenari M

NIH has modified its policy regarding the receipt of resubmissions (amended applications).See Guide Notice NOT-OD-18-197 at https://grants.nih.gov/grants/guide/notice-files/NOT-OD-18-197.html. The impact/priority score is calculated after discussion of an application by averaging the overall scores (1-9) given by all voting reviewers on the committee and multiplying by 10. The criterion scores are submitted prior to the meeting by the individual reviewers assigned to an application, and are not discussed specifically at the review meeting or calculated into the overall impact score. Some applications also receive a percentile ranking. For details on the review process, see http://grants.nih.gov/grants/peer_review_process.htm#scoring.

MEETING ROSTER

The roster for this review meeting is displayed as an aggregated roster that includes reviewers from multiple MD Special Emphasis Panels of the 2020/08 NIMHD
for the 2020/08 council round.
This roster for MD is available at:
http://public.era.nih.gov/pubroster/Reports?DOCTYPE=SEP&DESFORMAT=PDF&AGENDA_SEQ_NUM_P=394034
